# An event based analysis of extreme rainfall and historical trend in southern Tamil Nadu

**DOI:** 10.1038/s41598-025-27240-1

**Published:** 2025-12-08

**Authors:** Bidare Phalanetra Deepthi, Kotigaanahalli Nanjundegowda Vishwanth, Mysore KeshavaRao Harikeerthan, Supraja Irukumati, Suresh Devaraj, Hulivahana Nagaraju Sowmya, Abhinav Wadhwa, Amudha Soundarrajan, Kalel Ahamed A., Parthiban Loganathan

**Affiliations:** 1 Dayananda Sagar Academy of Technology & Management, Bengaluru, 560082 India; 2 Centre for Remote Sensing and Geoinformatics, Sathyabama Institute of Science and Technology, Chennai, 600119 India; 3 Department of Civil Engineering, Dayananda Sagar College of Engineering, Bangalore, 560078 India; 4 Discovery Partners Institute, The Grainger College of Engineering, University of Illinois Urbana-Champaign, Chicago, 60606 USA; 5 Department of Computer Science (Artificial Intelligence and Data Science), Dr. SNS Rajalakshmi College of Arts and Science, Coimbatore, Tamil Nadu 641049 India; 6 Central Water Commission, New Delhi, Delhi 110066 India; 7 Department of Engineering Mechanics, KTH Royal Institute of Technology, 11428 Stockholm, Sweden

**Keywords:** Extreme rainfall, Flood mitigation, Climate change, Southern Tamil Nadu, Rainfall variability, Environmental impact, Climate sciences, Environmental sciences, Hydrology, Natural hazards

## Abstract

Floods are a recurring natural hazard in India, and their frequency and severity are escalating due to the compounded effects of climate change and anthropogenic pressures. This study investigates the catastrophic flooding that occurred in the southern Tamil Nadu districts of Kanyakumari, Tenkasi, Tirunelveli, and Thoothukudi in December 2023, triggered by extreme rainfall associated with Cyclone Michaung. Analysis of long-term rainfall data from 1901 to 2023 reveals a significant increase in rainfall variability, seasonality, and the frequency of extreme events, particularly during the October–December northeast monsoon period. The rainfall recorded on December 17, 2023, exceeded the 100-year return period in Tirunelveli and Thoothukudi, and the 50-year return period in Kanyakumari and Tenkasi indicating a statistically rare and hydrologically severe event. Seasonality indices (PCI, PCD, SI) and onset–withdrawal trends further highlight a shift toward concentrated, high-intensity rainfall episodes and longer monsoon durations. HAND-based flood inundation modeling, validated using ground truth points, delineated over 150 km^2^ of affected area, particularly along the Thamirabarani basin. The research emphasizes the need for proactive flood management in the coastal districts of Thoothukudi, Tirunelveli, Tenkasi, and Kanyakumari. Key strategies include creating flood inundation maps through dynamic hydrological–hydraulic modeling, establishing early warning systems, implementing sustainable land-use practices, and developing green infrastructure. Long-term climate adaptation measures are also crucial, such as climate modelling for future rainfall predictions, investing in climate-resilient infrastructure, and educating communities about flood preparedness.

## Introduction

Floods are on the rise globally, triggered by climate change, urban sprawl, and human actions^1^. The year 2023 witnessed devastating floods worldwide, causing billions in economic losses and displacing millions^2^. In Asia, floods and storms dominated disaster reports in 2022, accounting for a staggering 83% of major events. These events impacted over 50 million people and tragically resulted in over 5000 deaths^3^. Notably, the World Meteorological Organization (WMO) reports that Asia’s temperature rise between 1991 and 2022 has doubled compared to the previous three decades (1961–1990)^3^. India faces a growing threat of floods due to climate change, according to the IPCC-AR6 (Intergovernmental Panel on Climate Change) reports. These reports predict more frequent and intense rainfall events, increasing flood risks^4^. This aligns with current trends, as India has witnessed a rise in floods across various regions, including Assam, Delhi, and Mumbai^5^. These devastating floods have caused widespread damage and displacement.

Floods have been a recurring natural disaster in India, affecting various regions and causing significant loss of life and property^6^. The history of floods in India is marked by several catastrophic events, influenced by the country’s diverse climate and topography^7^. River basins, particularly the Ganges and Brahmaputra, have been prone to seasonal flooding due to monsoon rains^8^. In 2023, a cloudburst in Himachal Pradesh unleashed sudden and devastating floods^9^. Just two years prior, in 2021, a breach in the Chamoli glacier triggered a massive glacial outburst flood^10^. The 2013 Kedarnath floods remain etched in memory for their sheer scale of destruction^11^. Even earlier events, like the 2007 Shimla floods^12^ and the 2005 Sutlej River flash floods^13^, stand as stark reminders of the region’s vulnerability. While the Himalayas witness heavy rain-induced floods and glacial lake outbursts, other parts of the country are also susceptible to flooding due to various factors such as monsoons, cyclones, and overflowing rivers^14^. Gujarat, for instance, experienced devastating floods in 2005 and 2017 due to excessive rainfall overflowing the Narmada and Tapi rivers. These events caused significant economic losses and disrupted the livelihoods of thousands^15^. Similarly, Maharashtra has borne the brunt of multiple floods. Mumbai and Pune faced catastrophic events in 2005, while Kolhapur, Sangli, and Satara districts witnessed devastating floods in 2019^16^. The state was hit again in 2021, with Mahad and Chiplun experiencing widespread flooding^17^. Kerala, known for its lush greenery, has been ravaged by floods in recent years. The state witnessed widespread flooding and landslides due to heavy rainfall in 2018, 2019, and 2021^18^. Chennai, the state capital, has been struck by frequent floods in 2015, 2021, and 2023, primarily attributed to cyclones. Additionally, districts like Cuddalore and Nagapattinam are frequently affected by floods^8^.

Southern states, particularly Southern Tamil Nadu regions are typically less prone to severe flooding compared to other parts of India^19^. While the northeast monsoon brought typical rainfall patterns to most of India, the southern Tamil Nadu districts of Thoothukudi and Tirunelveli experienced a record-breaking event during December 2023^20^. Data from the Regional Meteorological Centre reveals that Kayalpattinam received a staggering 94.6 cm of rainfall, the highest ever recorded for the northeast monsoon season^21^. This amount is nearly 90% of the district’s typical annual rainfall, highlighting the extreme intensity of the rainfall.

Climate change is increasingly recognised as a major driver of rising flood risks^22^. Scientists are investigating the underlying physical processes that exacerbate these extreme events, with global warming identified as a key reason. Rising temperatures intensify atmospheric moisture and disrupt regional rainfall patterns, thereby amplifying the frequency and severity of floods^23^. Alongside climatic drivers, human-induced factors such as rapid urbanisation, land-use changes, and poor planning have also significantly contributed to recent flood disasters.

In India, floods wreak havoc across diverse landscapes, with their causes and impacts varying by region^24^. In mountainous regions, heavy rainfall quickly overwhelms steep river channels, while coastal areas face the dual threat of intense precipitation and storm surges^25^. Conversely, flat terrains are more vulnerable to waterlogging and prolonged inundation due to inadequate drainage and unregulated urban growth. These regional differences underscore the importance of context-specific flood mitigation strategies^26^.

Heavy rainfall remains the primary trigger of floods across India’s varied physiographic zones. The Himalayan and Western Ghats regions are particularly prone to extreme rainfall, with some events exceeding 100 cm in a single day (as shown in Table 1). A recent event in Kayalpattinam, which received a whopping 94.6 cm of rain on December 17th, 2023, during the northeast monsoon. This amount of rainfall is typically associated with severe cyclones. The closest comparisons to Kayalpattinam’s downpour are the 116.8 cm recorded in Aminidivi, Lakshadweep on May 6th, 2004, and the 104.9 cm received at Vihar Lake in Mumbai on July 27th, 2005. Notably, the Lakshadweep event occurred alongside a cyclone passing near the islands, while Mumbai’s intense rainfall happened during the southwest monsoon, a weather system significantly larger than India’s northeast monsoon.

**Table 1:** Highest 24-h rainfall events in India.

Date	Place	State	Rainfall (cm)
June 14, 1876	Cherrapunji	Meghalaya	104
September 11, 1877	Jowai	Meghalaya	102
November 14, 1992	Kakkachi	Tamil Nadu	97
June 16, 1995	Cherrapunji	Meghalaya	156
May 06, 2004	Aminidivi	Lakshadweep	117
July 27, 2005	Ambarnath	Maharashtra	101
July 27, 2005	Vihar Lake	Maharashtra	105
June 17, 2022	Mawsynram	Meghalaya	10

Despite typically receiving moderate rainfall during the monsoons (averaging 328.4 mm and 443.3 mm for southwest and northeast monsoons, respectively), Tamil Nadu’s southern districts faced a catastrophic flooding event in December 2023. Intense downpours lashed the districts of Thoothukudi, Tirunelveli, Tenkasi, and Kanyakumari, leaving over 7500 people stranded, primarily in Thoothukudi and Tirunelveli. This disaster caused significant economic losses. This study investigates the rainfall patterns over these four districts and comprehensively analyse the flooding in southern Tamil Nadu. The southern Tamil Nadu region encompassing Tirunelveli, Thoothukudi, Kanyakumari, Virudhunagar, and Ramanathapuram districts has experienced an intensification of flood hazards over the past century, with a marked increase in frequency and severity in the last two decades. Historically, the region’s flood regime has been influenced by both the southwest and northeast monsoons, with the latter (October–December) being the primary contributor to extreme rainfall events. This hydrometeorological vulnerability is compounded by the physiography of the region, including short, flashy river systems such as the Thamirabarani, Vaippar, and Gundar, which respond rapidly to mesoscale convective systems and cyclonic disturbances in the Bay of Bengal. Recent flood events including the catastrophic December 2023, January 2024 floods highlight the region’s growing exposure to high-intensity rainfall. Triggered by a stalled easterly wave and enhanced moisture convergence over the Gulf of Mannar, districts such as Thoothukudi and Tirunelveli received over 500 mm of rainfall in less than 72 h, overwhelming historical drainage networks and breaching nearly 60 water storage structures, including eroded tanks and aged masonry weirs.

In urban centers like Srivaikuntam and Palayamkottai, flash flooding was exacerbated by unregulated land use changes, encroachment of historical floodplains, and insufficient stormwater conveyance systems. Satellite-based flood mapping using Sentinel-1 SAR data confirmed inundation extents exceeding 150 km^2^ in southern Thoothukudi alone. The structural failures of several check dams and the overtopping of the Tamirabarani river embankments underscore the increasing inadequacy of legacy flood management infrastructure under altered rainfall regimes. Hydrologically, the December 2023 event was characterized by return periods exceeding 100 years at several IMD rainfall stations, suggesting a statistically significant departure from historical norms. Furthermore, district-level vulnerability assessments post-2023 floods reveal systematic gaps in flood preparedness, particularly in low-income peri-urban zones. The interplay of aging infrastructure, poor catchment management, and inadequate early warning systems necessitates urgent scientific and policy interventions. Adaptive strategies must include basin-scale hydrological modeling, dynamic reservoir operation protocols, rehabilitation of traditional tank systems, and stringent floodplain zoning. Without such integrated approaches, the region’s flood risk will likely continue to escalate under a warming climate and growing developmental pressure. This study investigates recent rainfall anomalies and their hydrological implications, aiming to comprehensively analyse the heavy flooding events in southern Tamil Nadu. The findings contribute to a growing body of evidence on compound climate risks and inform future adaptation planning in monsoon-dominated semi-arid regions.

## Materials and methods

**Fig. 1:**
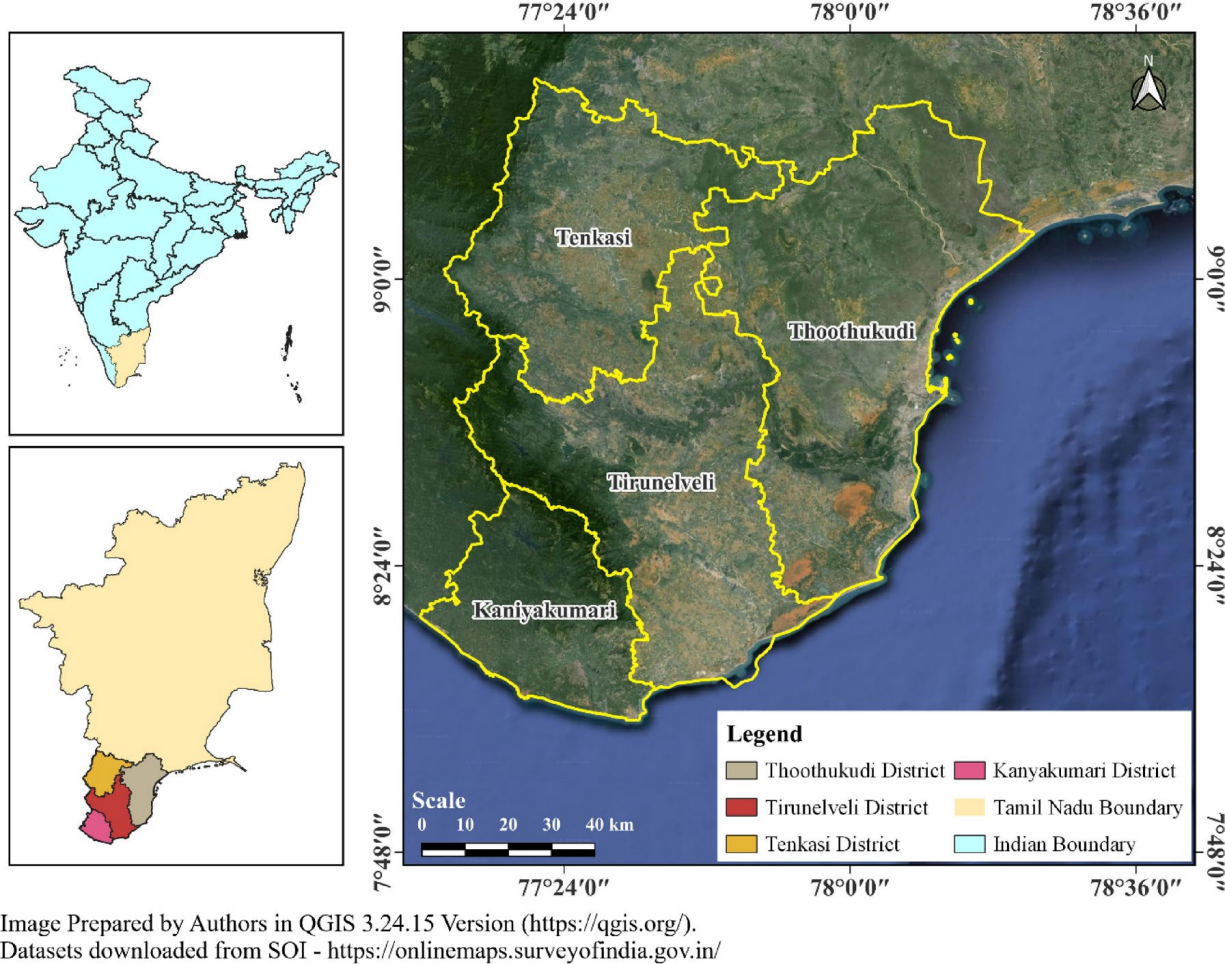
Geographical location of the study area.

The present study is focused on the southernmost part of Tamil Nadu, encompassing the districts of Thoothukudi, Tirunelveli, Tenkasi, and Kanyakumari. Geographically, the region extends between 77.10° E–78.39° E Longitude and 8.08° N–9.42°N Latitude, covering an area of about 13210.51 km^2^, representing a diverse landscape that transitions from the coastal plains of the Bay of Bengal to the elevated terrains of the Western Ghats. Each of these districts holds unique physiographic, climatic, and socio-economic characteristics. Thoothukudi district, situated along the eastern coastline, is an important industrial and maritime hub with a major port, extensive fisheries, and salt pans, making it highly exposed to cyclonic storms, storm surges, and coastal flooding. Tirunelveli district is largely agricultural, sustained by the Tamiraparani River irrigation system, and represents the semi-arid plains of southern Tamil Nadu. Tenkasi district, carved out from Tirunelveli, lies along the foothills of the Western Ghats and is rich in biodiversity, forest cover, and water resources, with its elevated terrain playing a critical role in orographic rainfall. Kanyakumari, the southernmost tip of the Indian peninsula, is characterized by a unique blend of mountainous and coastal environments, making it ecologically sensitive as well as a prominent cultural and tourism centre. Climatically, the study area is strongly influenced by both the southwest and northeast monsoons, with rainfall variability across districts due to altitudinal and coastal influences. This combination of physiographic diversity and climatic variability makes the region highly vulnerable to multiple hydro-climatic hazards, including floods, droughts, and cyclones, thereby underscoring the significance of studying its environmental dynamics for effective resource and disaster management. The average annual rainfall in these districts is relatively low, ranging from 110.71 to 727 mm. Additionally, the Tamirabarani River provides a vital source of freshwater for agriculture. However, in December 2023, these very districts faced devastating floods, highlighting the growing unpredictability of weather patterns. The geographical location of the study area is presented in Fig. 1.

**Fig. 2:**
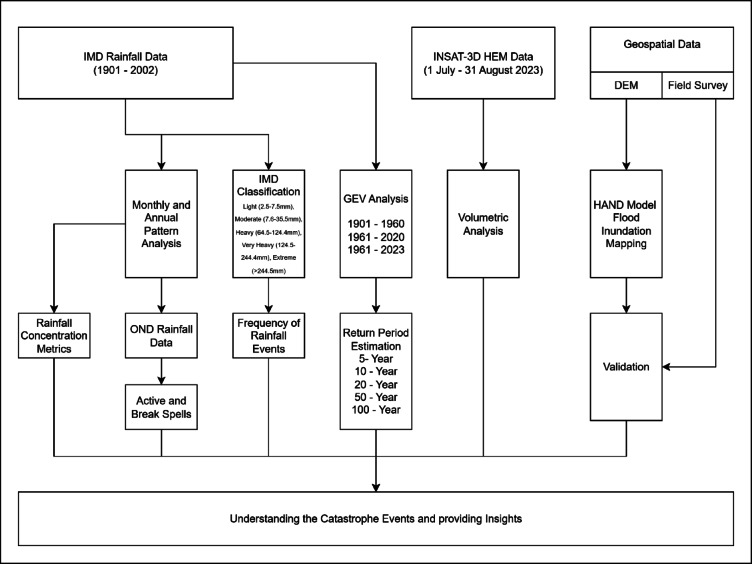
Methodological workflow adopted in the present study.

Long-term daily rainfall data from the Indian Meteorological Department (IMD) with a 0.25-degree spatial resolution was obtained for the period 1901 to 2023. This data provides historical context for rainfall patterns in the region. Sub-hourly rainfall data for December 2023 (covering the flood period) acquired using the Indian National Satellite System (INSAT) using the 3D Hydro-Estimator Method (HEM) is obtained from Meteorological and Oceanographic Satellite Data Archival Centre (MOSDAC). This high-resolution data allows for a detailed analysis of the specific rainfall patterns during the floods. To provide a detailed insights of the disaster event over the southern part of Tamil Nadu, the present study involves several analysis including, analysing the historical IMD rainfall datasets, rainfall concentration metrics, seasonal rainfall progression and rain spell analysis, frequency analysis, flood mapping and performing a volumetric analysis over the considered regions. Figure 2 presents the methodological work flow adopted in the present study.

### Rainfall concentration metrics

To assess the temporal concentration and irregularity of rainfall, the Precipitation Concentration Degree (PCD), Precipitation Concentration Index (PCI), and the Walsh & Lawler Seasonality Index (SI were computed. PCD measures the degree to which annual rainfall is concentrated in a particular part of the year (i.e. the non-uniformity of the intra-annual precipitation distribution)^27^ and is computed using Eq. 1. It was introduced by Zhang and Qian (2003) in the context of studying droughts and floods in China^28^.

1 $$PCD{ = }\left| {\frac{1}{P}\sum\limits_{i = 1}^{12} {P_{i} } .e^{{j\theta_{i} }} } \right|$$

where $p_{i}$ is rainfall month i,P is the total annual rainfall, θ_i_ = 2πi/12, and the magnitude of the resultant vector reflects seasonal asymmetry. High PCD (close to 1) denotes rainfall dominance in a single month.

The PCI (Precipitation Concentration Index) is a simpler metric that quantifies the variability of monthly precipitation totals relative to the annual^28^ and derived by Eq. 2. It was originally proposed by Oliver (1980) as a “powerful indicator” of temporal rainfall distribution. Essentially, PCI increases when a few months account for a large fraction of annual rainfall, and it is low when precipitation is evenly spread out.

2 $$PCI{ = }\frac{{\sum\limits_{i = 1}^{12} {p_{i}^{2} } }}{{\left( {\sum\limits_{i = 1}^{12} {p_{i} } } \right)^{2} }}{\text{ x 100}}$$

PCI values below 10 indicate uniform distribution, while values above 20 reflect extreme seasonal concentration.

The Walsh & Lawler Seasonality Index (1981) is a classic metric used to characterize the intra-annual distribution of rainfall and classify climate regimes by rainfall pattern^29^ and is computed using Eq. 3. SI essentially compares each month’s rainfall to the uniform monthly mean and sums the deviations. It was originally developed to describe and compare rainfall regimes (e.g. distinguishing an equatorial ever-wet climate from a strongly seasonal monsoonal climate). A higher SI means rainfall is confined to a shorter part of the year (more seasonal), while a low SI means rainfall is spread more evenly across months.

3 $$SI{ = }\frac{1}{P}\sum\limits_{i = 1}^{12} {\left| {p_{i} - \frac{P}{12}} \right|}$$

### Seasonal rainfall progression and rain spell analysis

To identify rainfall build-up and decay phases, the onset and withdrawal dates of the OND (October–December) rainfall season were extracted using a dynamic percentile threshold method. For onset detection, daily rainfall exceeding the 70th percentile over 3 consecutive days was used; for withdrawal, values falling below the 25th percentile for 5 consecutive days signaled termination. Furthermore, the active and break spells based on daily rainfall relative to district-specific OND thresholds. Active spells correspond to rainfall above the 75th percentile (Q75), while break spells fall below the 25th percentile (Q25), with durations ≥ 2 days.

### Rainfall extremes and GEV frequency analysis

Rainfall frequency analysis is performed using Generalized Extreme Value (GEV)^30^ to understand the frequency of rainfall over the four districts. The GEV probability distribution function (PDF) provides the likelihood of observing a particular rainfall intensity, while the cumulative distribution function (CDF) gives the probability that rainfall does not exceed a given threshold. This analysis utilized the IMD 0.25-degree gridded rainfall data spanning two periods: 1901 to 1960 and 1961 to 2023 (including the flooded duration). The return period analysis was stratified into two intervals, 1901–1960 and 1961–2023, to capture both climatological and data quality considerations. The GEV Distribution encompasses three parameters, encompassing Gumbel (Type I), Frechet (Type II), and Weibull (Type III) extreme value distributions. The cumulative distribution function of the GEV is expressed through Eq. 4.

4 $$F(x;\mu ,\sigma ,\xi ) = \exp \left\{ { - \left[ {1 + \xi \left( {\frac{x - \mu }{\sigma }} \right)} \right]^{ - 1/\xi } } \right\},{\text{ for }}1 + \xi \left( {\frac{x - \mu }{\sigma }} \right){ > 0}$$

where $\mu$, $\sigma$, $\xi$ represents location, scale, and shape parameters respectively. The distribution function (PDF), obtained by differentiating the CDF, describes the probability density associated with each rainfall value as expressed in Eq. 5.

5 $$f(x;\mu ,\sigma ,\xi ) = \frac{1}{\sigma }\left[ {1 + \xi \left( {\frac{x - \mu }{\sigma }} \right)} \right]^{{ - \frac{1}{\xi } - 1}} \exp \left\{ { - \left[ {1 + \xi \left( {\frac{x - \mu }{\sigma }} \right)} \right]^{{ - \frac{1}{\xi }}} } \right\},{\text{ for }}1 + \xi \left( {\frac{x - \mu }{\sigma }} \right){ > 0}$$

Statistically, the location parameter identifies the centre of the data’s distribution on the horizontal axis, scale controls out the distribution of data, and shape describes the data clusters around the centre. In flood risk studies, the CDF is crucial because it links rainfall intensity to return periods. In addition, the flood inundation extent is mapped using the Height Above the Nearest Drainage (HAND) model.

## Results

The coastal districts of Thoothukudi, Tirunelveli, Tenkasi and Kanyakumari in Tamil Nadu, India, are least prone to flooding due to heavy rainfall. These regions receive rainfall during the northeast monsoon (October–November) and southwest monsoon (June–September), with extreme rainfall events becoming more frequent and intense in recent years. The low-lying, flat coastal plains are particularly susceptible to inundation during heavy rains, and rivers like the Thamirabarani, Pazhayar, and Nambiyar often overflow, exacerbating the flood risk. Notable floods include the 1978 deluge in Tirunelveli’s Thamirabarani basin, the devastating 2006 cyclone-induced floods in Thoothukudi, the 2015 northeast monsoon floods in Kanyakumari, and the severe flooding in December 2023 across all four districts, which prompted extensive rescue operations and caused widespread damage.

### Analysis of rainfall

**Fig. 3:**
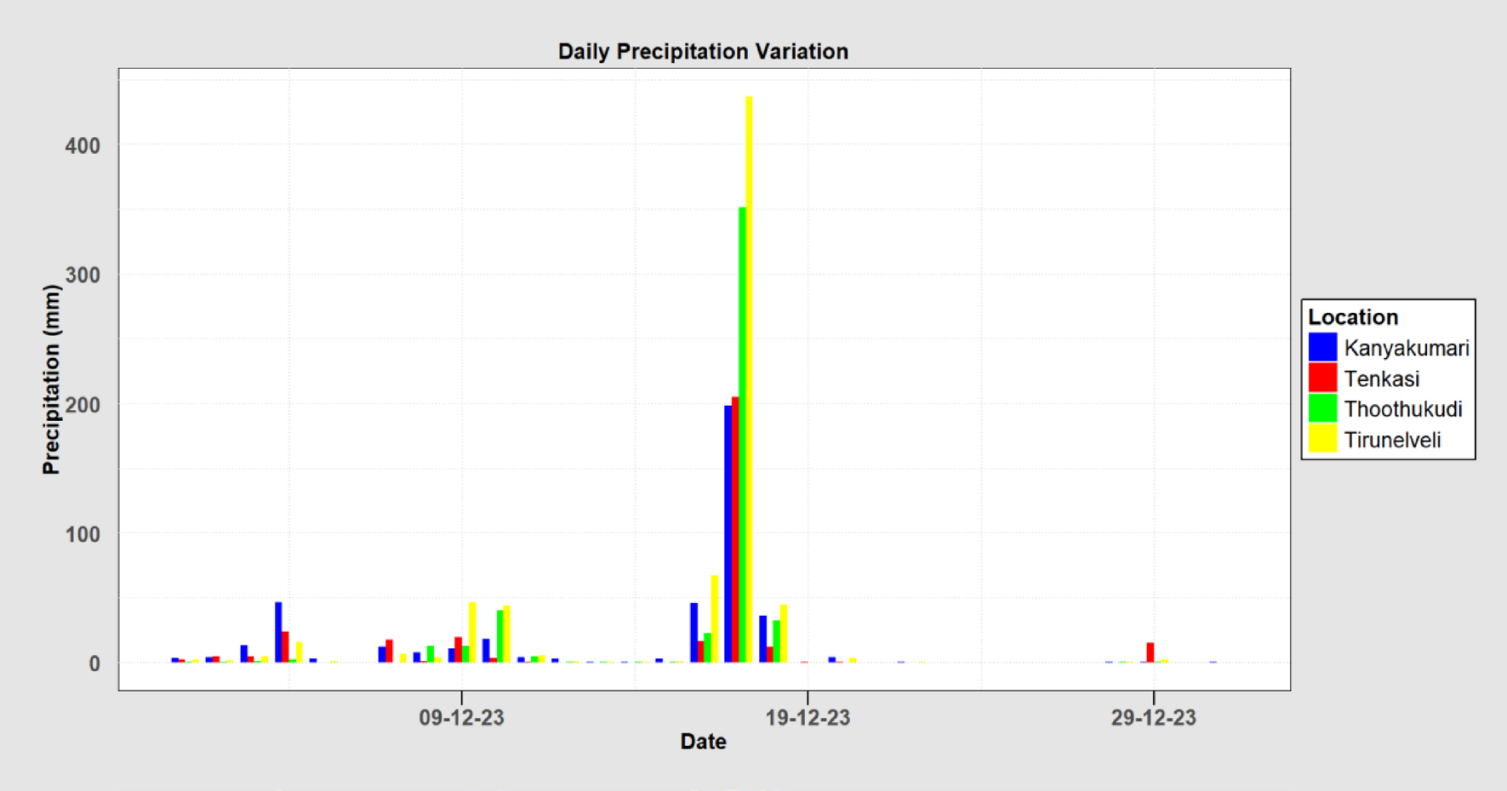
December 2023 rainfall in the study area.

**Fig. 4:**
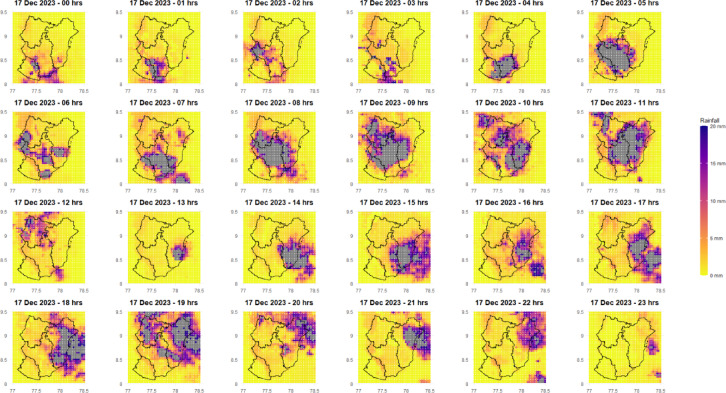
December 17, 2023 rainfall in the study area.

Data from the India Meteorological Department (IMD) reveals exceptionally high rainfall in Kanyakumari, Tenkasi, Thoothukudi, and Tirunelveli districts of Tamil Nadu on December 17, 2023. This analysis indicates one of the most intense rainfall events in recent history for these districts. Tirunelveli received the highest amount of rainfall (436.87 mm), followed by Thoothukudi (351.35 mm), Tenkasi (204.81 mm), and Kanyakumari (198.34 mm). As illustrated in Fig. 3, the daily rainfall pattern for these districts shows a concentration of rainfall on December 17th. This intense rainfall event, attributed to Cyclone Michaung, is believed to be the cause of the flash floods that occurred in the region. Figure 4 presents the rainfall distribution over the study area during the peak rainfall period (December 17, 2023). From the figure, it is clearly evident that the four districts received huge intensity of rainfall spread all over the districts, with Tirunelveli and Thoothukudi receiving the maximum rainfall.

**Fig. 5:**
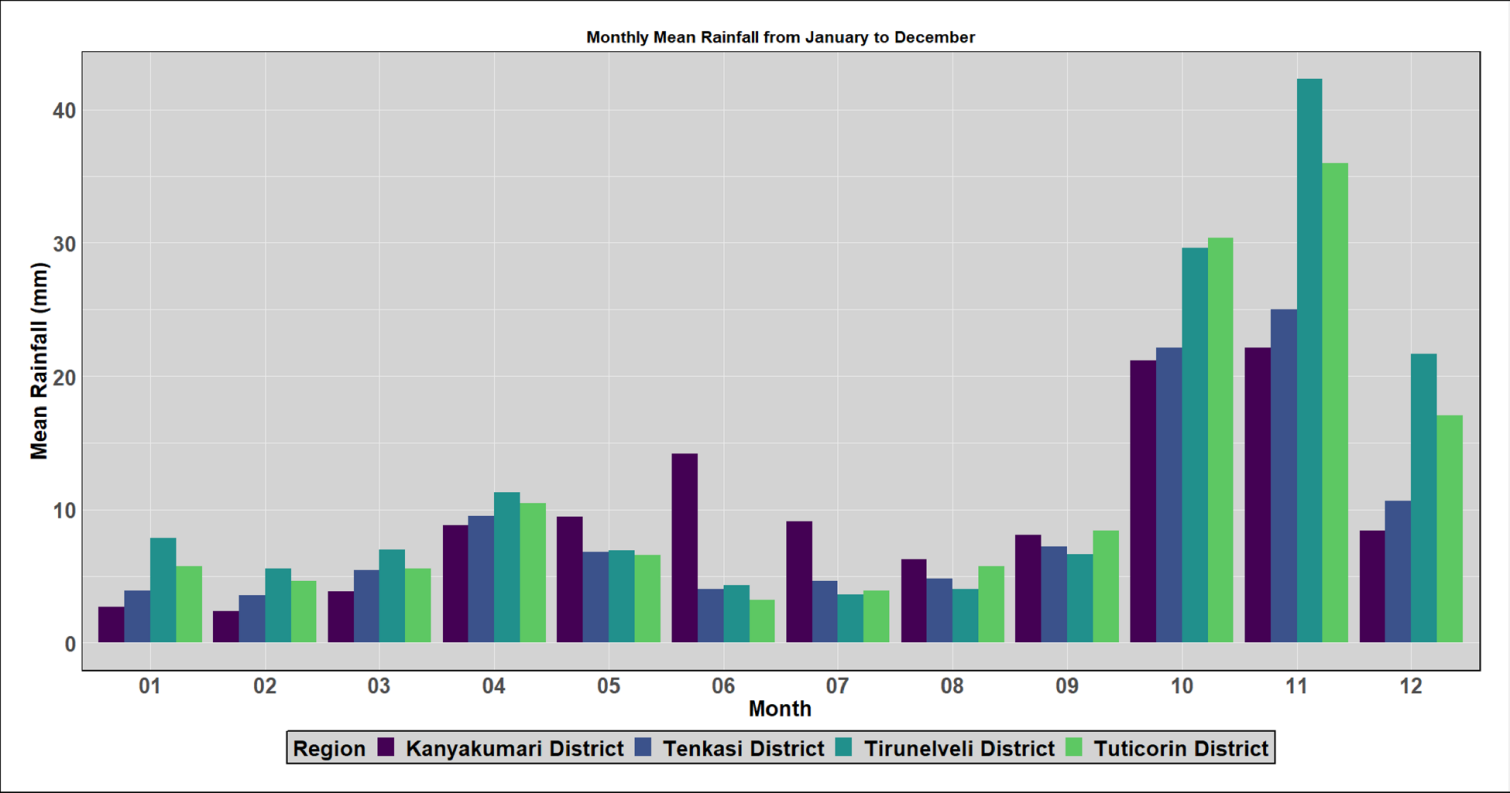
Monthly average rainfall from 1901 to 2023.

**Fig. 6:**
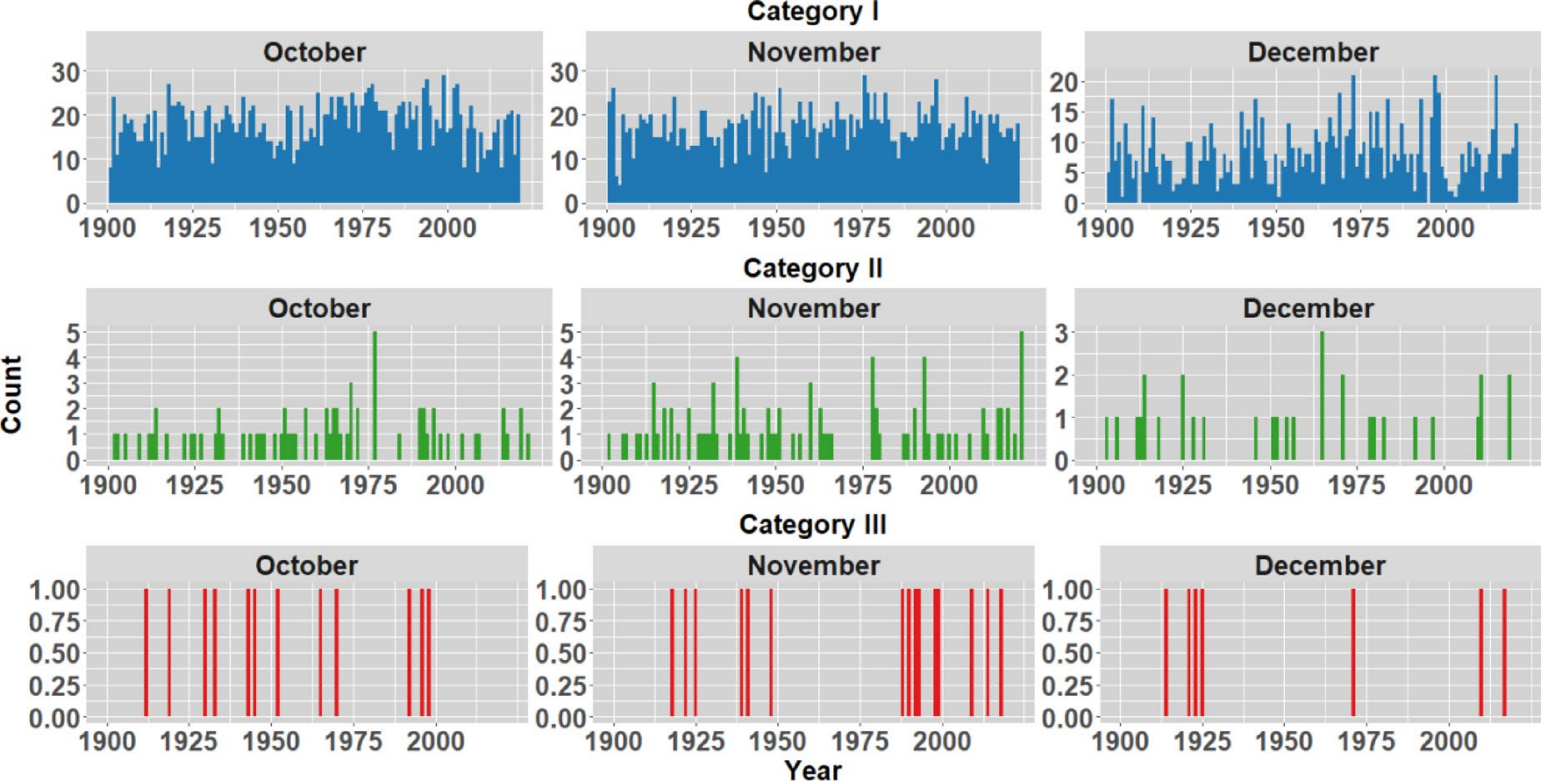
Frequency of rainfall events from 1901 to 2023 over Kanyakumari district.

**Fig. 7:**
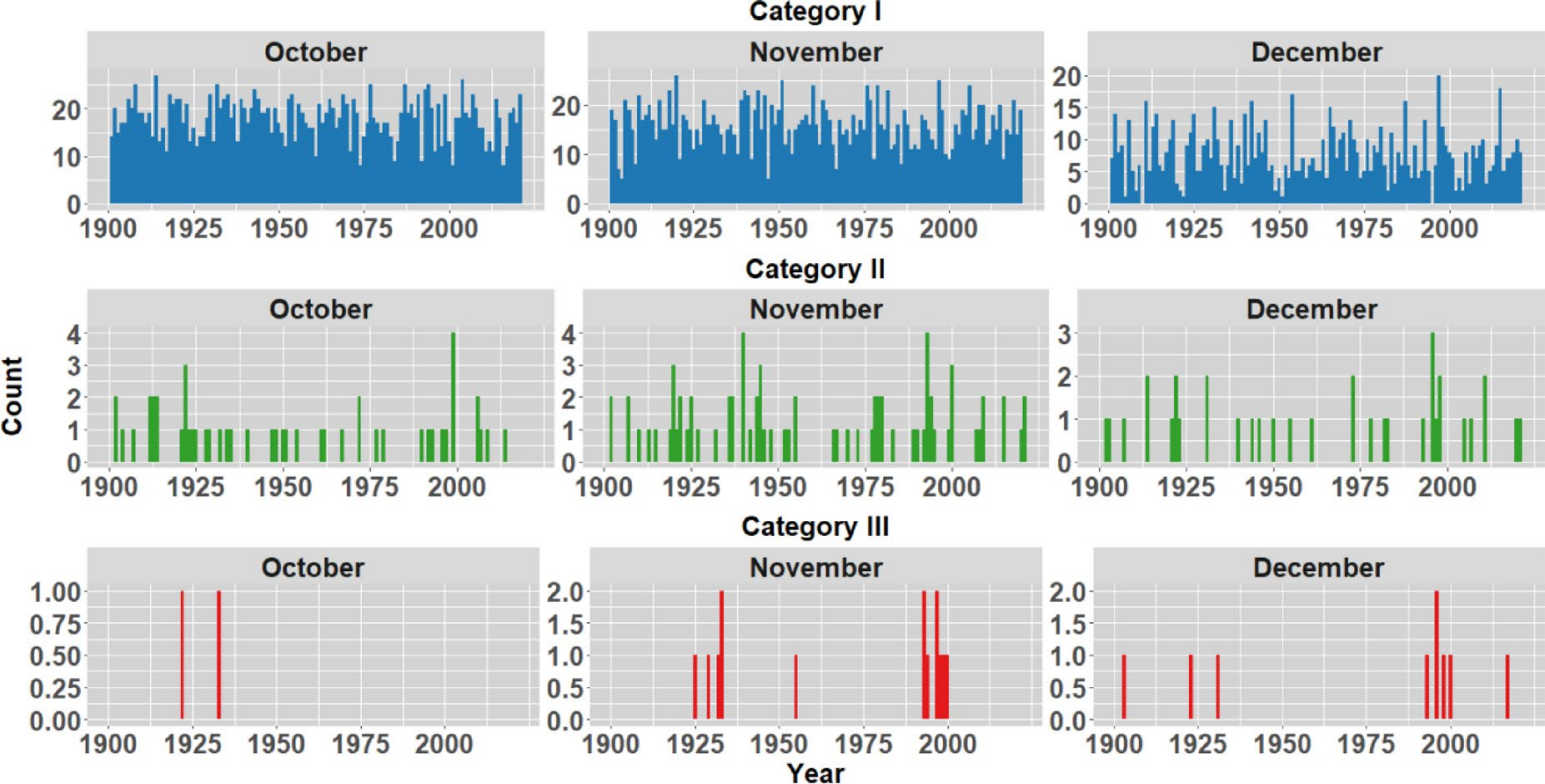
Frequency of rainfall events from 1901 to 2023 over Tenkasi district.

**Fig. 8:**
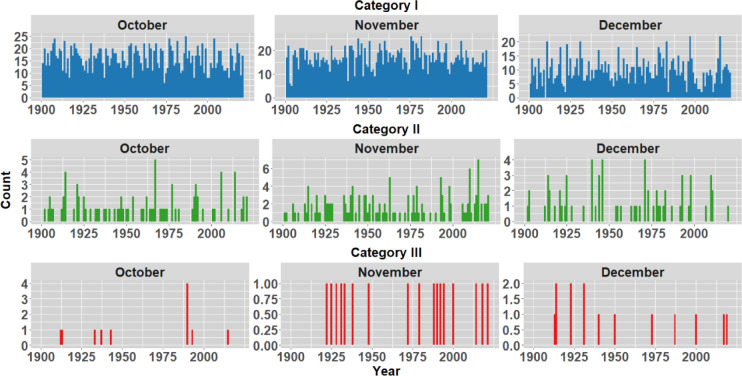
Frequency of rainfall events from 1901 to 2023 over Tirunelveli district.

**Fig. 9:**
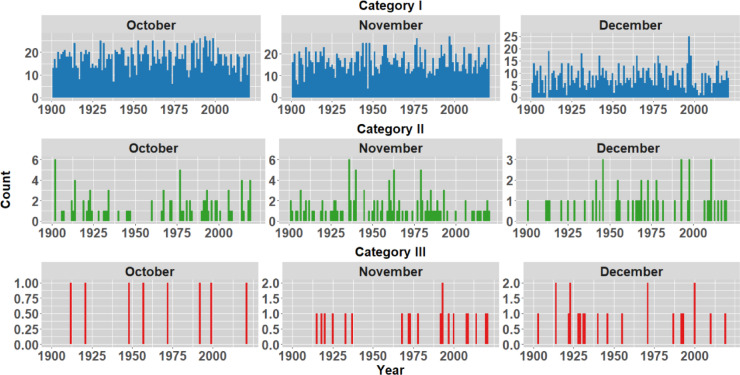
Frequency of rainfall events from 1901 to 2023 over Thoothukudi district.

To analyse the historical context of the extreme events in the districts opted in the study, IMD rainfall data spanning 1901 to 2023 is examined to understand average monthly rainfall patterns. The monthly analysis indicates a significant influx of rainfall primarily in October, November and December as shown in Fig. 5. In order to understand the frequency of rainfall occurrences within the districts, the rainfall during these months has been categorized into three distinct groups, as illustrated in Table 2 (Classification as per IMD standards). Figures 6, 7, 8 and 9 illustrates the frequency of rainfall from the year 1901 to 2023 over the districts Kanyakumari, Tenkasi, Tirunelveli and Thoothukudi districts respectively. Analysing the rainfall over Kanyakumari district, it is found that the district receives moderate rainfall and the district rarely experiences extreme rainfall. Similarly, Tenkasi and Thoothukudi district also periodical rainfall whereas the occurrence of extreme rainfall events is very less. Considering Tirunelveli district, in a span of 10 years, 6 extreme events were identified indicating the increase in the frequency of extreme rainfall over the district. Though the district experiences frequent extreme rainfall events in the month of November and December, the region is not flooded as the region is not highly affected by the antecedent rainfall or soil moisture.

**Table 2:** Classification of IMD rainfall in the present study.

Daily rainfall (mm)	IMD classification	Classification grouped in the present study
0	No rain	–
0.1–2.4	Very light rain	
2.5–7.5	Light rain	Category I
7.6–35.5	Moderate rain	
35.6–64.4	Moderate heavy rain	
64.5–124.4	Heavy rain	Category II
124.5–244.4	Very heavy rain	Category III
> 244.5	Extreme rain

**Fig. 10:**
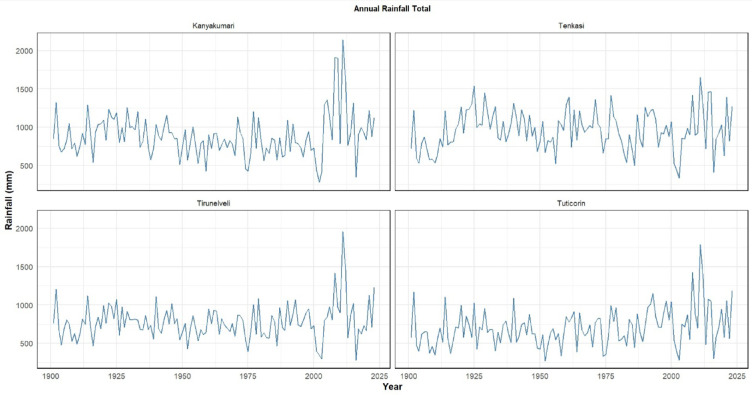
Annual rainfall received by the districts.

The analysis of long‐term annual rainfall totals across the four southern Tamil Nadu districts reveals both spatial and temporal contrasts in precipitation patterns as shown in Fig. 10. Kanyakumari consistently receives higher annual rainfall often exceeding 1000 mm and occasionally peaking beyond 1800–2000 mm. The rainfall series shows strong interannual variability but without a pronounced long‐term trend, although the period after 2005 stands out with multiple anomalously high rainfall years. Tenkasi experiences more moderate rainfall, generally within 800–1500 mm annually, with comparatively fewer drought years than the drier districts. However, it also shows heightened variability in recent decades, with pronounced wet years after 2000. Tirunelveli displays the greatest variability, fluctuating widely between 400 mm and nearly 2000 mm. Extended dry periods are interspersed with extreme wet years, particularly the exceptional peak around 2005–2010. This alternating pattern of deficit and excess reflects its vulnerability to both droughts and floods. Thoothukudi which remains the driest, typically ranging between 400 and 1000 mm/year. Post 2000s, an increase in rainfall extremes has been witnessed, with totals occasionally exceeding 1200 mm an unusual occurrence for this district.

The rainfall patterns across the four districts indicate that the region is experiencing increasing rainfall extremes, characterized by longer periods of drought interspersed with intense wet years. These shifts in precipitation patterns are consistent with broader regional and global trends in climate change, which are driving more extreme and unpredictable weather events^31^. Particularly, the years following 2000, especially 2005–2010 and 2023, show a significant rise in both the frequency and intensity of extreme rainfall events, leading to a growing risk of floods and other climate-related hazards.

**Fig. 11:**
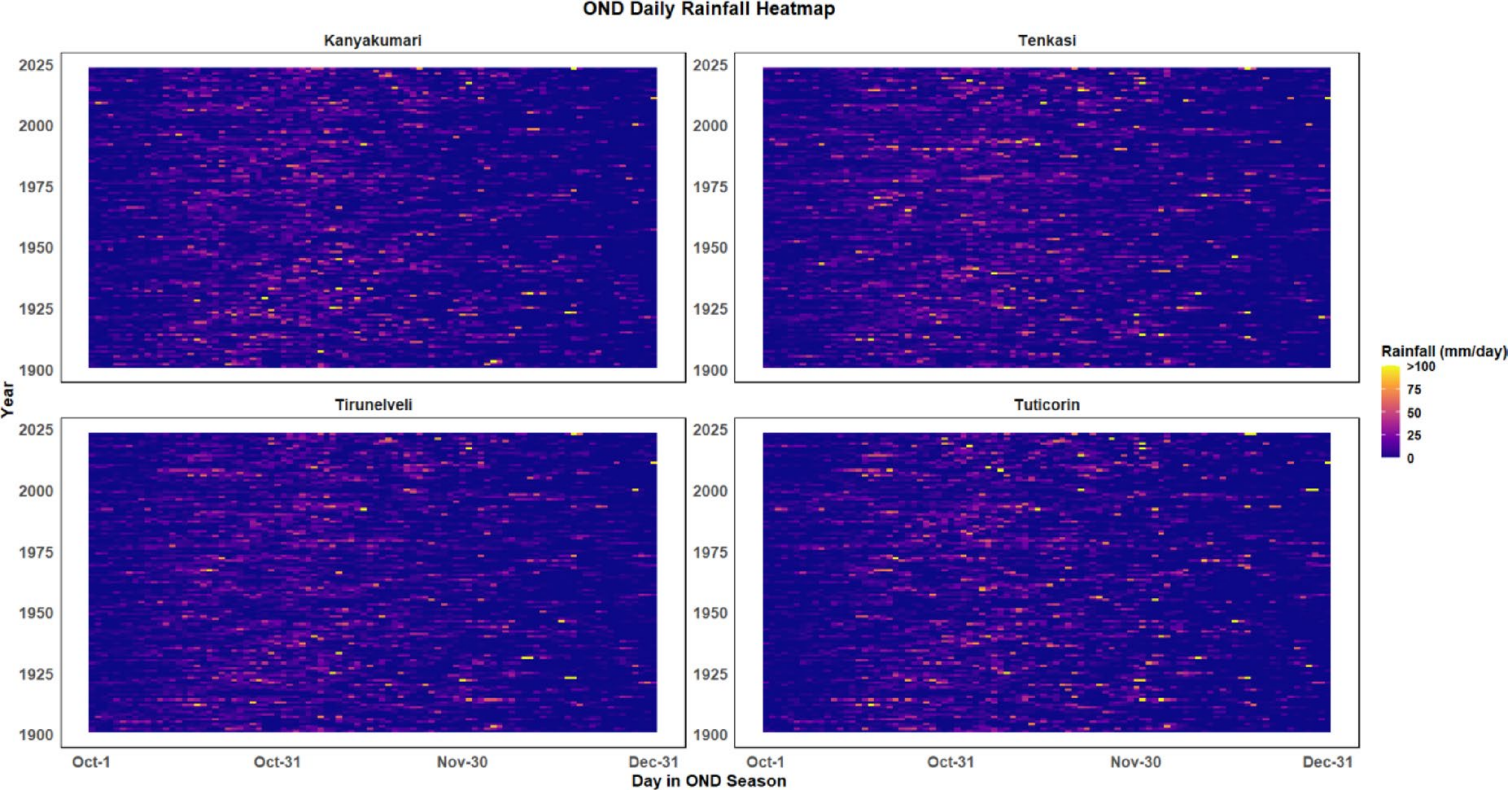
OND daily rainfall heatmap.

A heatmap representing the daily rainfall data during the October–November–December (OND) season across four districts in Southern Tamil Nadu is presented in Fig. 11. The vertical axis represents the years, and the horizontal axis indicates the days within the OND season, ranging from October 1 to December 31. The colour gradient reflects the intensity of rainfall, with purple representing minimal rainfall and yellow to red corresponding to higher rainfall amounts exceeding 100 mm/day.

Kanyakumari exhibits a frequent occurrence of intense rainfall, especially towards the end of the OND season, primarily in November and December, as highlighted by the yellow and red sections. This suggests that Kanyakumari is more prone to heavy rainfall events during this period, possibly linked to cyclonic disturbances and monsoonal fluctuations. In contrast, Tenkasi also shows significant rainfall but with fewer extreme events compared to Kanyakumari, indicating a generally less intense rainfall distribution. Tirunelveli experiences similar patterns, with most rainfall events occurring towards the end of the OND season, but with less frequent high-intensity rainfall, signifying a more moderate rainfall regime. Lastly, Tuticorin displays a relatively even rainfall distribution throughout the OND season, with fewer extreme rainfall events compared to the other districts, suggesting that while the district receives moderate rainfall, it is less prone to intense, extreme weather events.

**Fig. 12:**
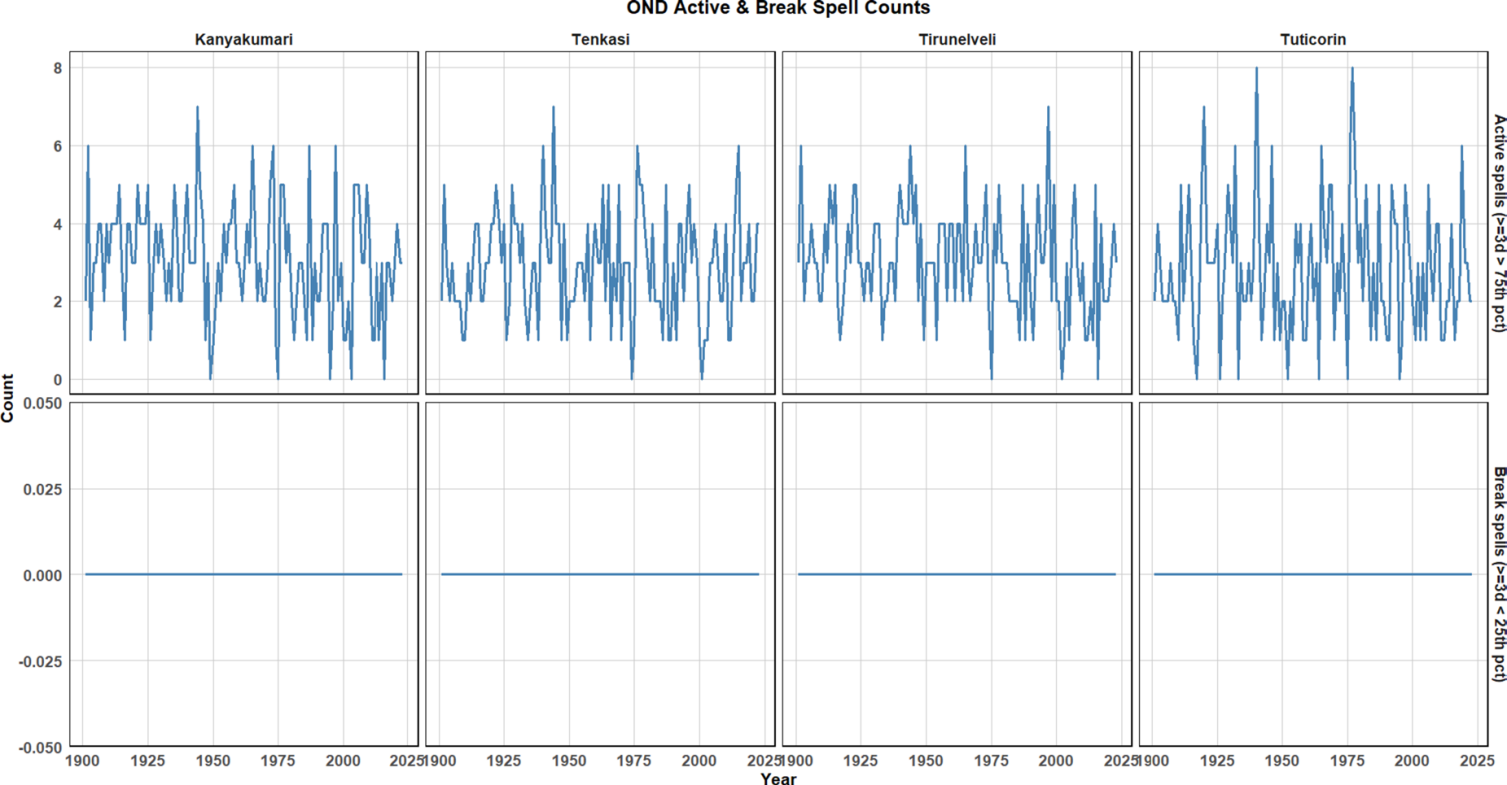
OND active and dry spells.

The analysis of the Active and Break Spell Counts during the OND season provides valuable insights into the seasonal rainfall patterns across the Southern Tamil Nadu districts. Figure 12 displays the active and break spells for each district, where active spells are identified by days of high rainfall above the 75th percentile (denoted as Q75), and break spells correspond to days with rainfall below the 25th percentile (denoted as Q25). The Active spell counts show fluctuations over the years, with some years exhibiting significantly higher counts. These spikes represent the increased frequency and intensity of rainfall events in recent decades, which aligns with broader global climate change trends. Specifically, the rise in active spells is indicative of more frequent extreme weather events, possibly driven by climate change, where the atmosphere holds more moisture, leading to heavier rainfall during specific seasons. This pattern suggests that southern Tamil Nadu is experiencing more frequent heavy rainfall episodes, particularly in the OND season, which traditionally contributes significantly to the region’s total annual rainfall. Break spells, which represent periods where daily rainfall falls below the 25th percentile of historical rainfall data is presented in Fig. 12. These dry spells indicate prolonged periods of below-average rainfall, often associated with drought-like conditions. However, the Break spell counts are zero across all years in all four districts. This lack of break spells suggests that there have been no substantial dry periods in the region that qualify as break spells based on the 25th percentile threshold. The region may generally experience consistent rainfall, especially during the OND season, making it less likely for rainfall to fall below the 25th percentile for extended periods. This absence of dry periods could also imply a reduction in the frequency of droughts or dry spells in recent decades, as the region has witnessed more frequent rainfall events and intense storms, particularly from cyclonic disturbances in the Bay of Bengal. The combined analysis of both active and break spells provides important insights into the changing rainfall dynamics in the region. The increased frequency of active spells, paired with the absence of break spells, points towards a shift in hydrological patterns where intense rainfall events are becoming more prevalent, while dry spells are either less frequent or shorter in duration. This pattern is a reflection of the broader impact of climate change, where rising temperatures lead to greater moisture retention in the atmosphere, subsequently increasing the potential for extreme rainfall during seasonal transitions.

**Fig. 13:**
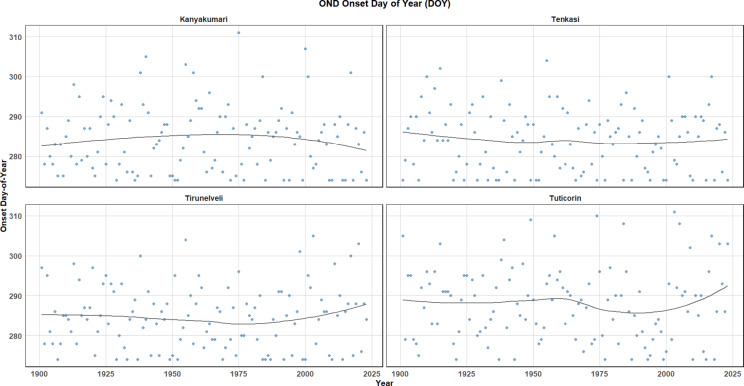
OND onset day of the year.

**Fig. 14:**
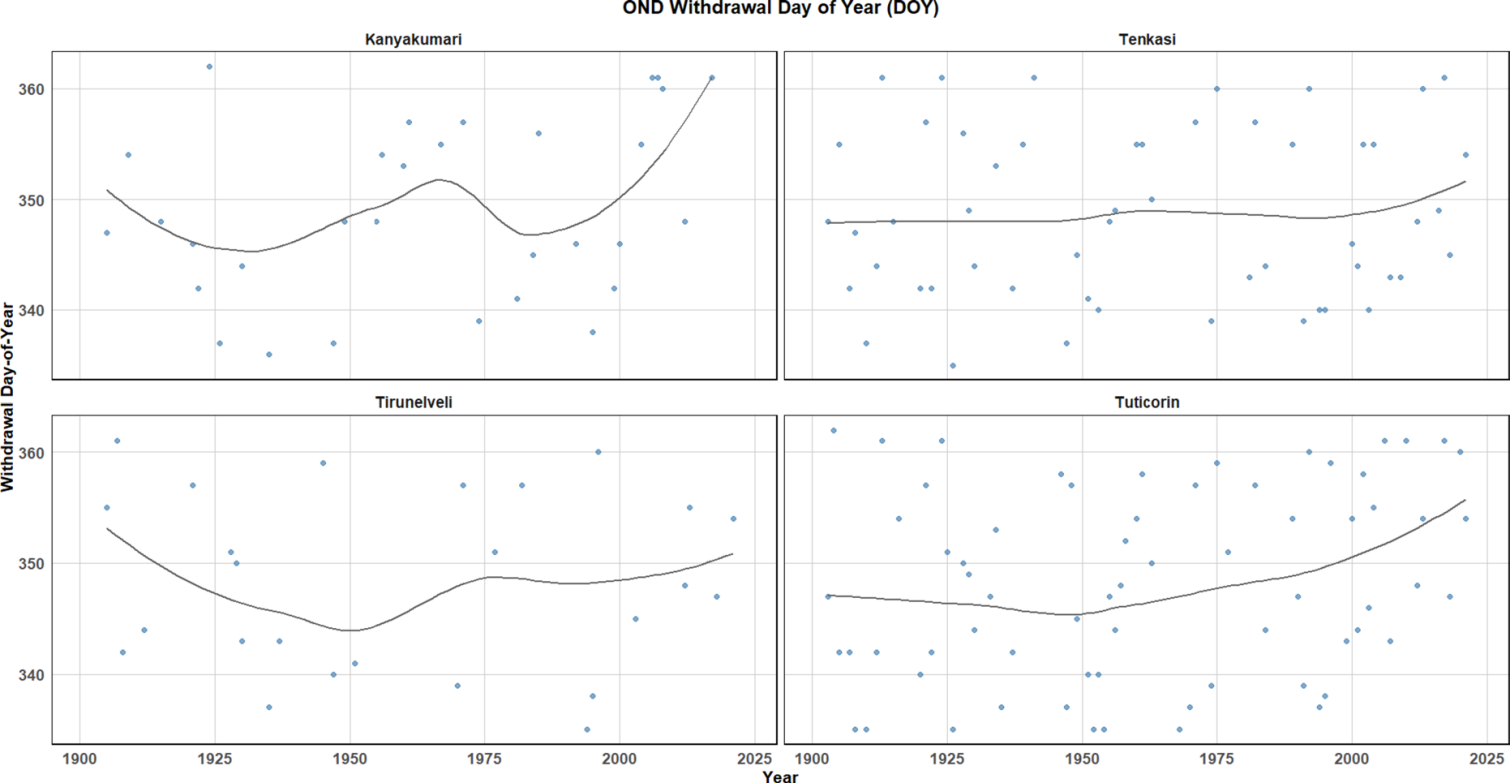
OND withdrawal day of the year.

The onset of the monsoon refers to the first significant rainfall event that marks the beginning of the monsoon season in a particular region. The withdrawal of the monsoon refers to the gradual cessation of the sustained monsoon rains. It marks the end of the monsoon season, characterized by a reduction in rainfall and the return to drier conditions. The onset and withdrawal define the duration of the monsoon and are crucial for understanding climatic patterns, agricultural cycles, and water resource management in regions that are heavily dependent on monsoon rains. Onset Day of Year (DOY) and Withdrawal Day of Year (DOY) for the OND season across four districts are presented in Figs. 13 and 14 respectively. These trends were analysed from 1900 to 2023, shedding light on the regional variations in the timing of the onset and withdrawal of the OND season. Figure 13, representing the Onset Day of Year shows a clear positive trend in Kanyakumari, with the onset of the season occurring early in recent decades. Tenkasi, on the other hand, displays a relatively flat trend, indicating stability in the timing of the monsoon onset. Conversely, Tirunelveli exhibits a negative trend, where the onset appears to be occurring earlier over time and recently there is a shift in the days which presents a delay. Thoothukudi (Tuticorin) demonstrates slight variations with no significant directional trend whereas recently the spell experiences a delay in the onset. The black trend lines in each panel represent smoothened curves that highlight these tendencies, showing a mix of gradual shifts across different regions.

Figure 14, which depicts the Withdrawal Day of Year, the trends reflect a more complex pattern. Kanyakumari shows a sinusoidal fluctuation, with the withdrawal day alternating between earlier and later over time. Tenkasi and Tuticorin both show gradual delays in the withdrawal day, suggesting a trend towards a later conclusion of the season. Tirunelveli, however, displays a negative trend, indicating that the withdrawal of the monsoon season has been occurring earlier in recent years and lately it experiences a delay. These results suggest significant regional differences in the timing of both the onset and withdrawal of the monsoon, which could be indicative of changing climate patterns in the region. Overall, the duration of the monsoon appears to be increasing in Kanyakumari and Tirunelveli, where both the onset and withdrawal are shifting towards later dates, while Tenkasi and Tuticorin show more stable patterns.

### Precipitation concentration degree (PCD)

**Fig. 15:**
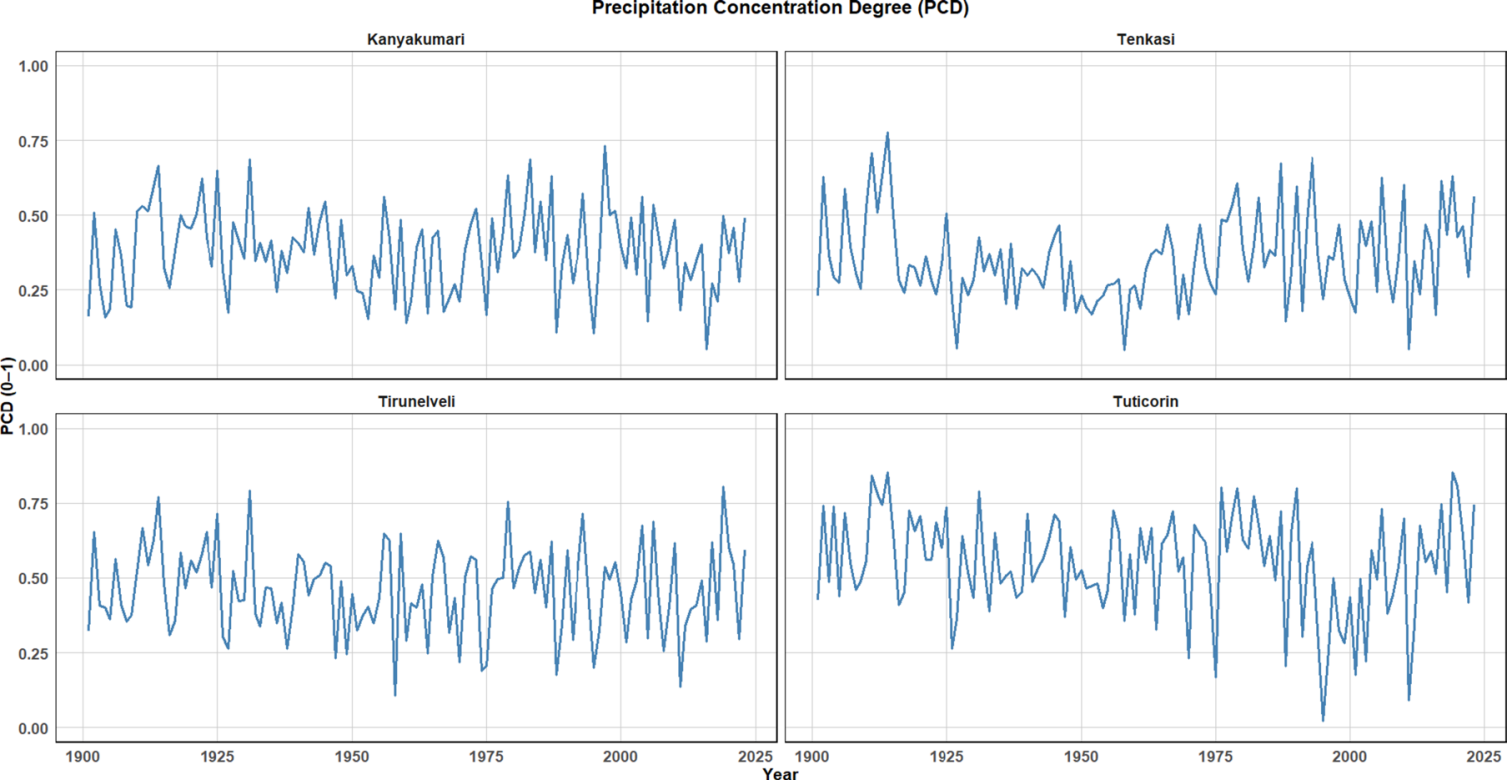
PCD over the four districts from 1901 to 2023.

The Precipitation Concentration Degree (PCD) is a valuable metric used to quantify the temporal concentration of rainfall within a given period, often reflecting how evenly or unevenly rainfall is distributed throughout the year. In the context, the PCD values for four districts, Kanyakumari, Tenkasi, Tirunelveli, and Tuticorin are plotted over the years, and these time series provide insights into the precipitation patterns in Southern Tamil Nadu as shown in Fig. 15.

The PCD time series for Kanyakumari shows notable fluctuations, with periods of higher and lower concentration. It indicates a varying rainfall distribution across different years, where the rainfall is more concentrated during the monsoon months, but it is spread out across the year in others. This suggests that the district experiences significant variability in rainfall concentration, which can impact agricultural planning and water resource management. Similar to Kanyakumari, Tenkasi’s PCD series shows cyclical variations, with the highest concentrations of rainfall occurring at certain points in the year, most likely aligning with the monsoon season. The year-to-year variation in PCD reflects the erratic nature of rainfall, suggesting a high dependency on the timing and intensity of the monsoon rains. Tirunelveli shows a relatively higher and more consistent PCD across the years, indicating that rainfall in this district is more evenly distributed throughout the year compared to the other districts. This could be due to a combination of factors such as regional climate patterns, topography, and local rainfall dynamics. A higher PCD here suggests that the region may be less prone to extremes like flash floods but could face challenges related to maintaining consistent water availability throughout the year, such as managing irrigation systems. The PCD values for Tuticorin reflect a pattern similar to that of Tenkasi and Kanyakumari, but with some distinct peaks. These peaks highlight years where rainfall was heavily concentrated, likely contributing to more extreme weather events like floods. The variability seen in the PCD values suggests that the region might be sensitive to climate fluctuations and could experience both dry spells and heavy rainfall within the same year, which can complicate water management strategies.

### Precipitation concentration index (PCI)

The Precipitation Concentration Index (PCI) is a metric used to assess the distribution of rainfall over time, specifically the degree of concentration or dispersion of precipitation within a defined period. It is calculated by comparing the total rainfall for each time period (e.g., year, month) with the total rainfall for the entire season. A higher PCI value indicates that rainfall is concentrated in fewer but more intense events, while a lower PCI value suggests a more evenly distributed rainfall pattern over the period.

**Fig. 16:**
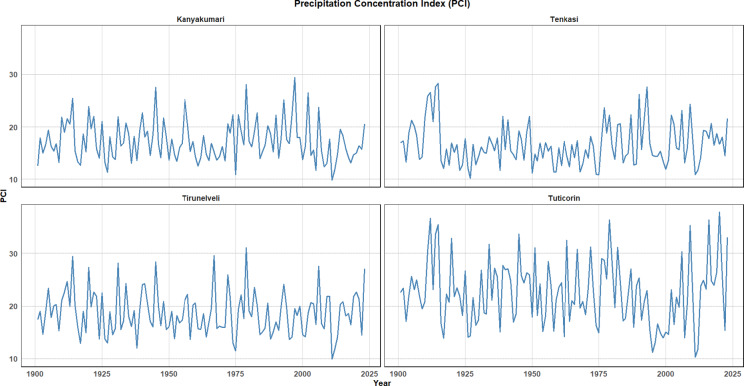
PCI over the four districts from 1901 to 2023.

Figure 16 illustrates the temporal trends in the Precipitation Concentration Index (PCI) for the four districts. The PCI trend for Kanyakumari shows moderate fluctuations, with a relatively steady range over the years, indicating that rainfall is somewhat concentrated, but the variability between years is less extreme. Tenkasi exhibits similar fluctuations to Kanyakumari but with slightly more pronounced peaks. The values suggest a relatively moderate concentration of rainfall, with occasional years showing more intense rainfall periods. The PCI for Tirunelveli demonstrates a notable increase in concentration in the recent decades, especially from the 2000s onwards. This suggests that the region may be experiencing more intense and less frequent rainfall events, which could have implications for water management and agricultural planning. In contrast to the other districts, Tuticorin shows more substantial variability in the PCI, with higher spikes indicating years with more concentrated rainfall events, particularly towards the recent years (2020–2025). This could suggest increased extremes in precipitation patterns.

Across all four districts, there is a general trend of increasing PCI in the most recent years, which implies more extreme rainfall events and possibly a shift towards more intense monsoons. This is particularly evident in districts like Tirunelveli and Tuticorin. The increasing concentration of rainfall could be associated with changing climate patterns, potentially linked to broader climatic shifts or local factors. The observed trends may have significant implications for flood risks, water resource management, and agricultural planning in these regions.

### Walsh-Lawler seasonality index (SI)

**Fig. 17:**
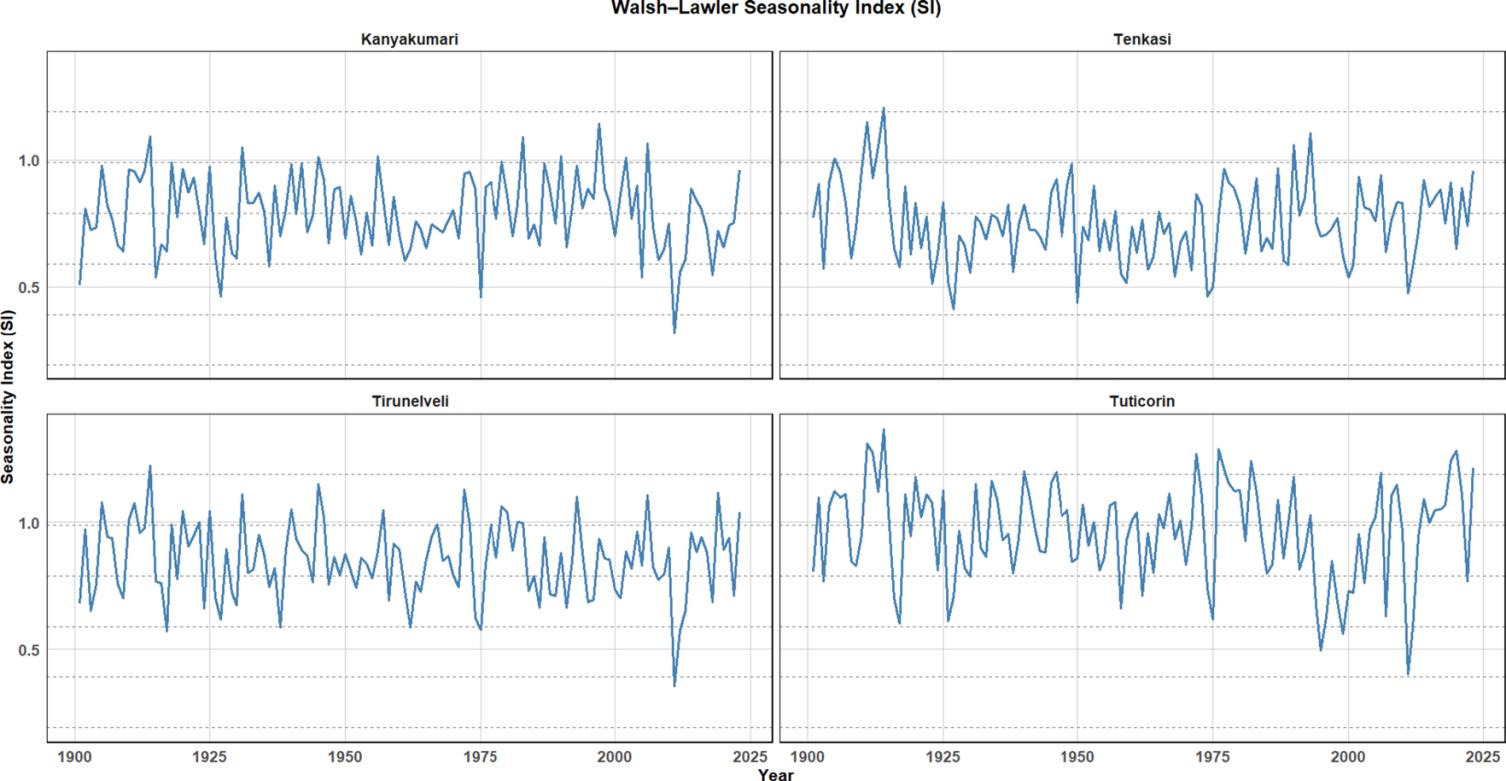
Walsh-Lawler seasonality index over the four districts from 1901 to 2023.

The Walsh-Lawler Seasonality Index (SI) is a widely used metric to assess the temporal distribution of precipitation across different seasons. It helps measure the variability and intensity of seasonal fluctuations in rainfall, where values closer to 1 indicate a well-defined seasonal pattern, and values closer to 0 suggest more uniform distribution of rainfall throughout the year. The SI provides valuable insights into the degree of seasonality of precipitation and can help assess drought vulnerability, agricultural patterns, and hydrological behavior in different regions. Figure 17 presents the Walsh-Lawler Seasonality Index (SI) for four districts spanning from the year 1900 to 2023. Analysing the SI, it is identified that Kanyakumari shows relatively consistent fluctuations indicating a moderate seasonality of rainfall with slight deviations throughout the period. This suggests that Kanyakumari experiences relatively stable seasonal patterns with some variability, likely influenced by both the southwest and northeast monsoons. Tenkasi demonstrates a similar seasonal trend to Kanyakumari but with slightly more pronounced variability. This could reflect a region influenced by more distinct dry and wet periods, possibly due to its proximity to the Western Ghats and complex topography, which may enhance rainfall during specific seasons. Tirunelveli presents a mix of moderate and sharp fluctuations in its SI. These fluctuations indicate more pronounced seasonal rainfall patterns, possibly due to the stronger influence of monsoon systems in this region. This behavior could be associated with the region’s agricultural cycles, where crops are sensitive to seasonal rainfall shifts. Tuticorin also shows distinct seasonal variations but with a more erratic trend compared to the other regions. This suggests that the region may be subject to unpredictable shifts in rainfall patterns, potentially indicating vulnerability to extreme weather events such as droughts or floods, particularly in coastal areas.

### Rainfall frequency analysis

**Fig. 18:**
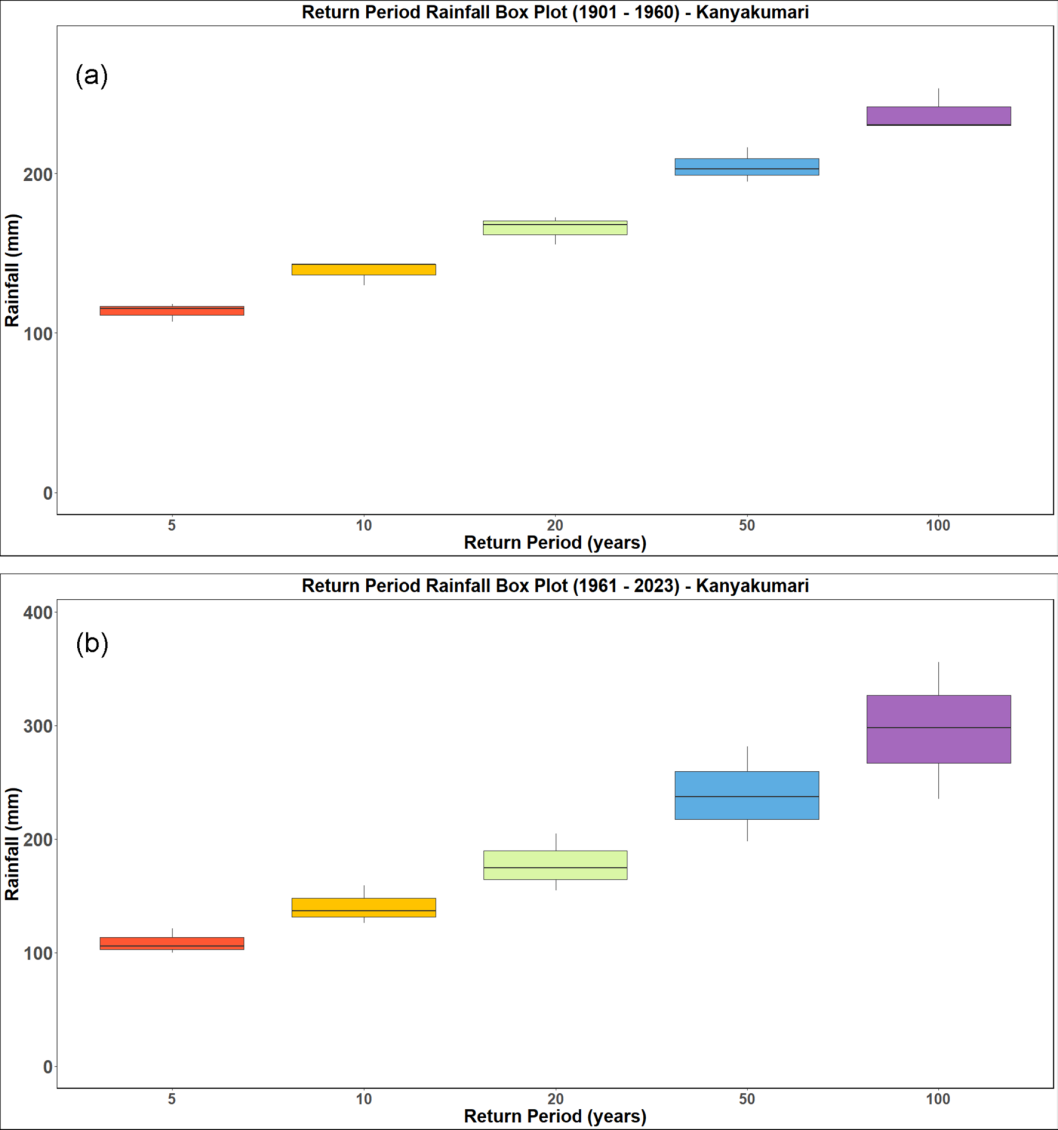
Rainfall return period for Kanyakumari district (**a**) 1901–1960 (**b**) 1961–2023.

**Fig. 19:**
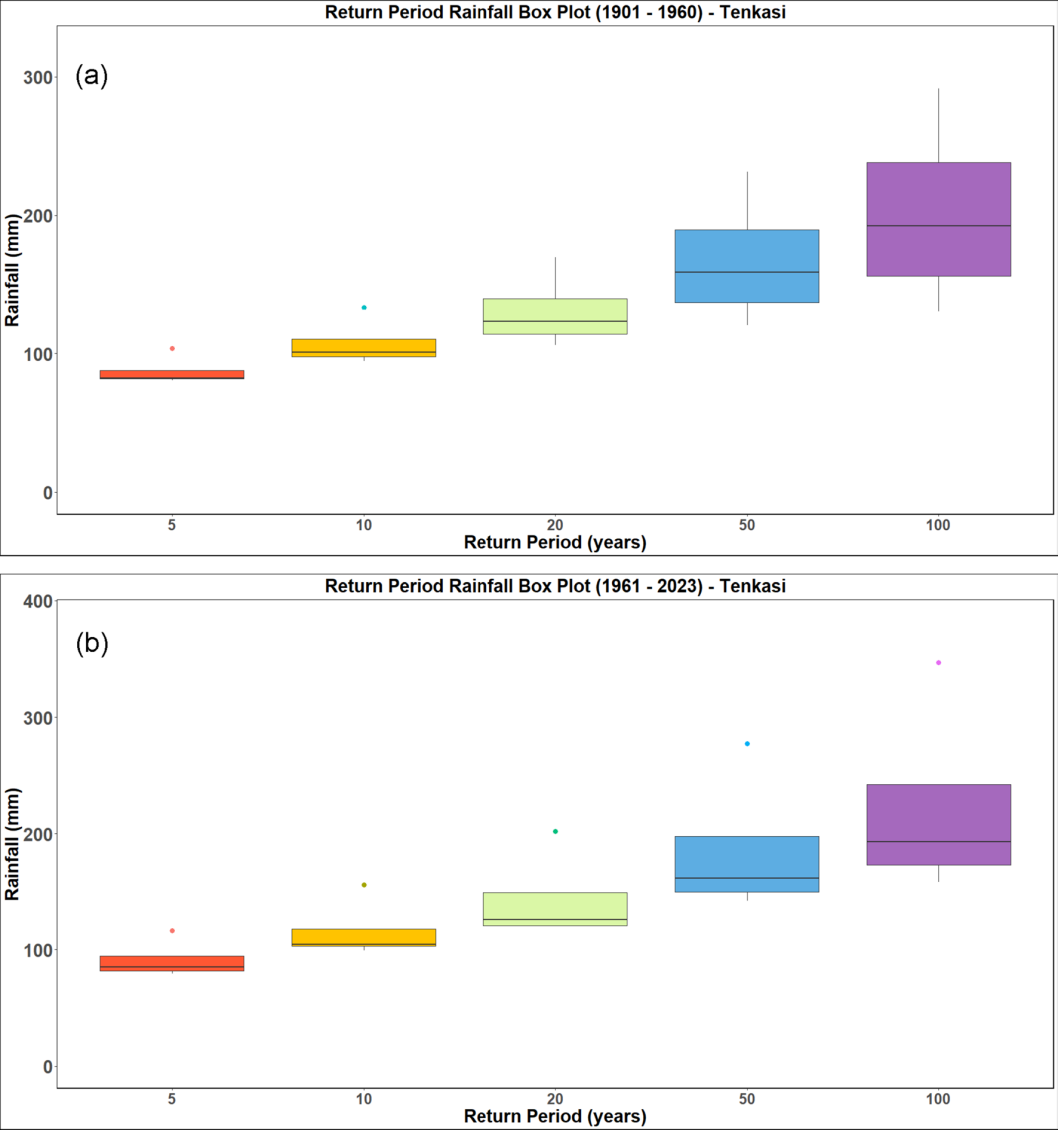
Rainfall return period for Tenkasi district (**a**) 1901–1960 (**b**) 1961–2023.

**Fig. 20:**
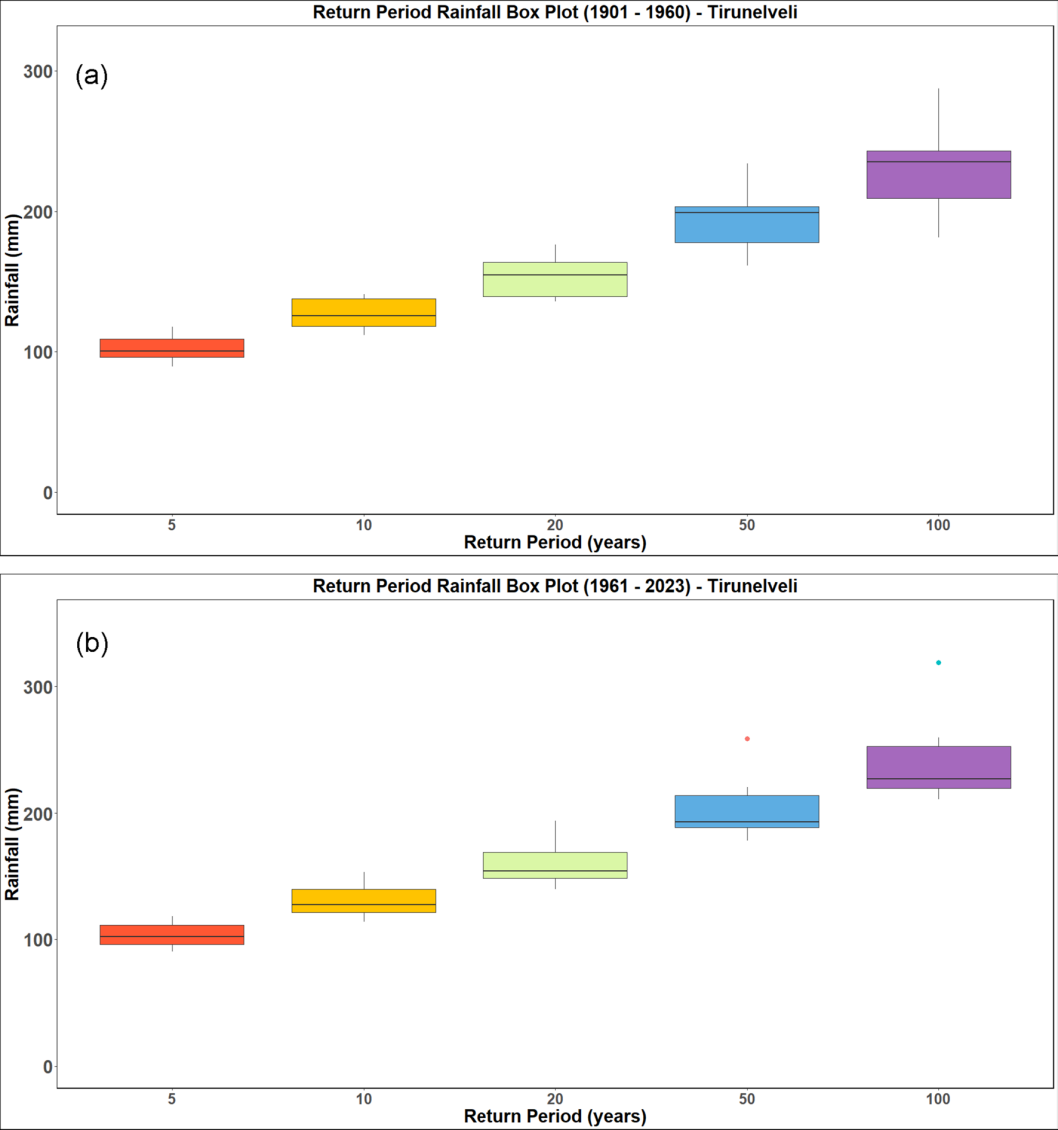
Rainfall return period for Tirunelveli district (**a**) 1901–1960 (**b**) 1961–2023.

**Fig. 21:**
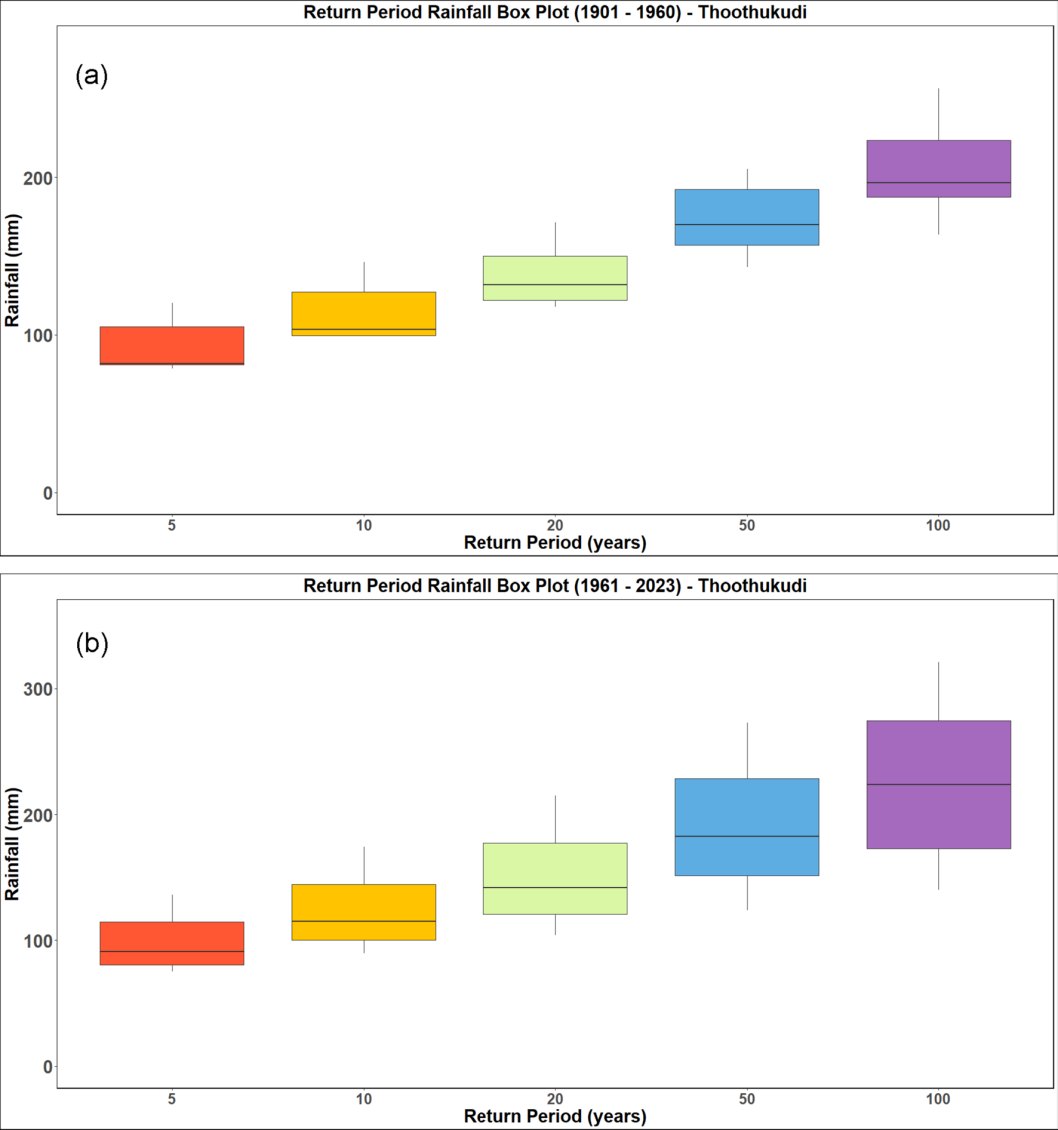
Rainfall return period for Thoothukudi district (**a**) 1901–1960 (**b**) 1961–2023.

The analysis is performed for a return period of 5, 10, 20, 50 and 100 years. The return period obtained by analysing the IMD datasets from 1901 to 1960 and 1961 to 2023 for the districts of Kanyakumari, Tenkasi, Thoothukudi and Tirunelveli districts and are presented in Figs. 18, 19, 20 and 21 respectively. Analysing the datasets the return period along with the intensity of rainfall is presented in Table 3.

**Table 3:** Rainfall return period over the districts (in mm).

Return period	Tirunelveli		Kanyakumari		Thoothukudi		Tenkasi	
	1901–1960	1961–2023	1901–1960	1961–2023	1901–1960	1961–2023	1901–1960	1961–2023
5	86.75	91.00	113.33	108.67	92.33	98.33	86.75	91.00
10	106.75	115.75	137.67	140.00	114.00	123.33	106.75	115.75
20	130.00	143.25	165.00	177.33	137.33	150.33	130.00	143.25
50	167.00	185.00	204.00	238.33	173.17	190.50	167.00	185.00
100	201.25	222.50	237.33	295.67	204.67	225.33	201.25	222.50

The results (summarized in Table 3) show a clear intensification in rainfall magnitudes associated with longer return periods, particularly in the post-1960 era. For instance, the 100-year return period rainfall increased from 201.25 to 222.50 mm in Tirunelveli and from 204.67 to 225.33 mm in Thoothukudi, indicating a growing likelihood of high-impact rainfall episodes. The December 17, 2023 rainfall recorded at 436.87 mm in Tirunelveli and 351.35 mm in Thoothukudi exceeded the modeled 100-year return period threshold, classifying it as a statistically rare and extreme hydrological event. Likewise, Kanyakumari and Tenkasi experienced rainfall corresponding to 50-year return periods, reinforcing the exceptional nature of this event across the region.

To further evaluate the temporal evolution of rainfall extremes, a Generalized Extreme Value (GEV) distribution was fitted for three time frames: 1901–1960, 1961–2020, and 1961–2023. The GEV parameters location (µ), scale (σ), and shape (ξ) (Table 4) offer insights into changes in average maxima, variability, and tail behaviour of the rainfall distribution. Analysis of the location parameter revealed a decreasing trend from 1901–1960 to 1961–2020 in Kanyakumari, Tenkasi, and Tirunelveli, suggesting a reduction in average extreme rainfall. However, in the most recent period (1961–2023), a reversal is observed, with values increasing in all districts, implying a resurgence in extreme rainfall in just the past few years. This increase is particularly notable between 2021 and 2023, aligning with observed rainfall anomalies and flood events.

**Table 4:** Shape, Slope and Location Parameters for the considered study area.

Location	Year	Location	Scale	Shape
Kanyakumari	1901–1960	71.07	25.39	0.15
	1961–2020	61.77	24.28	0.24
	1961–2023	62.39	25.13	0.27
Tenkasi	1901–1960	55.36	18.24	0.18
	1961–2020	50.81	22.42	0.11
	1961–2023	51.29	23.23	0.17
Tirunelveli	1901–1960	62.40	23.20	0.18
	1961–2020	59.40	25.56	− 0.02
	1961–2023	60.38	26.46	0.17
Thoothukudi	1901–1960	56.12	22.15	0.16
	1961–2020	57.34	24.08	0.02
	1961–2023	58.74	25.07	0.15

The scale parameter, which reflects rainfall variability, showed a consistent upward trend in Tenkasi, Tirunelveli, and Thoothukudi across the three periods, while Kanyakumari exhibited a U-shaped trend initial decline followed by a rebound. This indicates increasing rainfall unpredictability, especially in the drier districts, potentially leading to abrupt and unmanageable flood scenarios. The shape parameter, which influences the tail behaviour and probability of rare extremes, also exhibited critical shifts. For example, Tirunelveli transitioned from a near-zero (–0.02) value in 1961–2020 to a positive value (0.17) in 1961–2023, signaling increased probability of extreme outlier events. Kanyakumari’s shape factor steadily rose from 0.15 to 0.27, confirming a growing risk of heavy rainfall extremes. Although Tenkasi experienced a dip during the mid-period, its risk profile also rebounded in recent years. Thoothukudi showed similar trends, with a mid-period dip followed by increasing risk in the most recent datasets.

Collectively, these trends confirm that rainfall extremes are becoming more frequent, more intense, and more spatially widespread across southern Tamil Nadu. While return periods offer a threshold-based understanding of risk, the GEV analysis captures the underlying statistical shift in the rainfall regime towards heavier tails, greater uncertainty, and sharper fluctuations. The December 2023 event aligns closely with these modeled trends, validating the analytical framework. It is important to note that while the return periods are calculated at district level, actual flood behavior may vary at sub-catchment scales due to topography, drainage, and land use. Nonetheless, this analysis reveals a statistically significant hydrological anomaly for the region and reinforces the need for urgent climate-adaptive flood management. These findings are in line with recent studies highlighting similar intensifications in hydrological extremes across South India^32–35^.

### Flood mapping

**Fig. 22:**
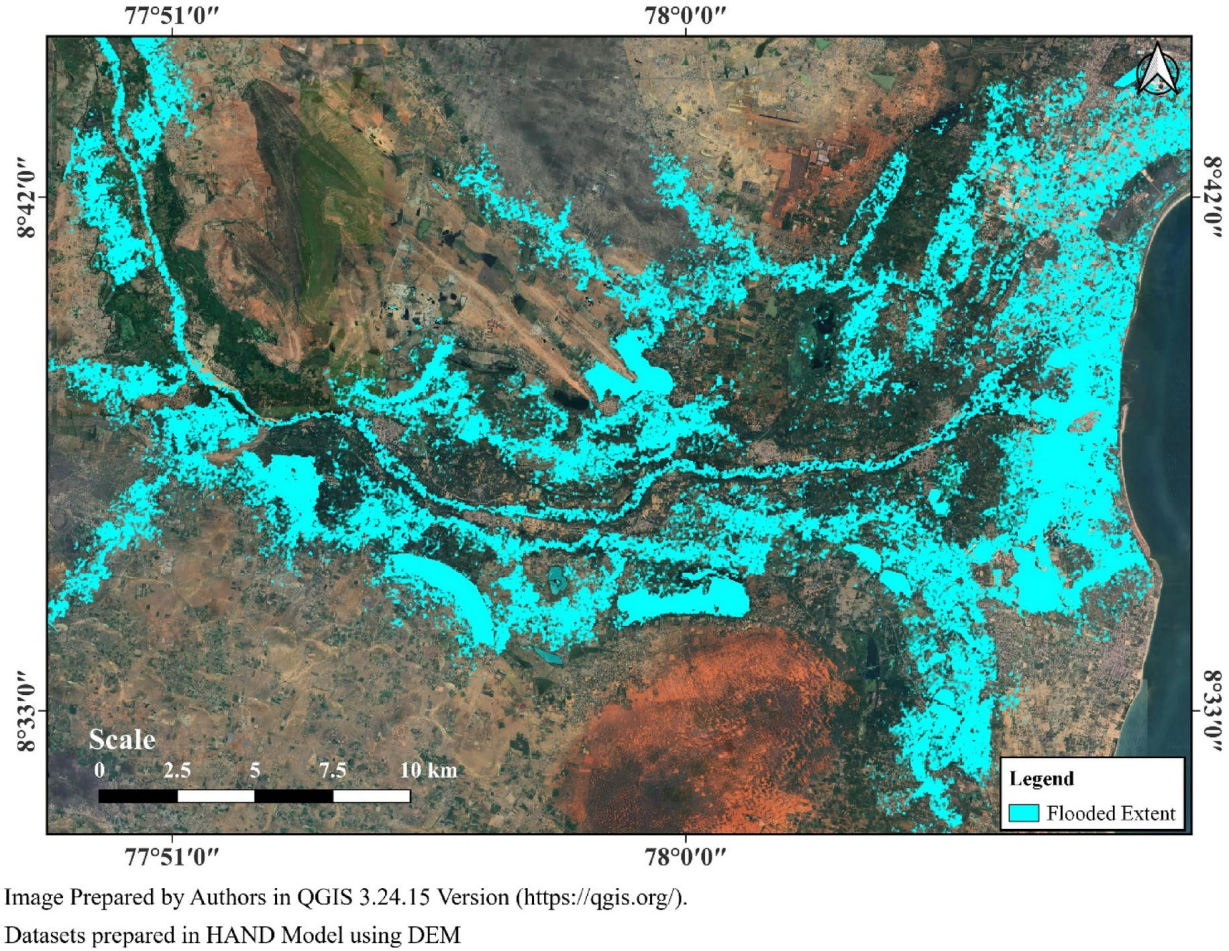
Flooded regions identified using HAND model.

Being highly affected by floods, identification of flood affected regions is essential to locate the villages and towns affected. Considering the limitations of the availability of discharge datasets, to develop a flood inundation map, HAND (height above nearest drainage) model is adopted with DEM as input. Firstly, the vertical distance of each land surface pixel from the nearest stream channel using DEM. These distances were further transformed into the calculation of flow direction and flow accumulation to delineate stream networks and identify drainage lines. Each pixel elevation is further compared to the downstream drainage cell to compute respective height above the channel. This resulted in the creation of a HAND raster, which represents the terrain vertical distance from drainage channels and highlights low-lying areas further providing flood inundation maps for the study area. The flood inundation map generated in presented in Fig. 22. To validate the same 96 ground truth points are collected through a field survey.

The accuracy assessment based on the 96 ground truth points (60 flooded and 36 non-flooded) shows that the classification performed well with high reliability. Out of the 60 flooded points, 53 were correctly identified as flooded (True Positives), while 7 were misclassified as non-flooded (False Negatives). All 36 non-flooded points were correctly classified (True Negatives), with no misclassification as flooded (False Positives). This resulted in an overall accuracy of 92.7%, reflecting strong agreement between HAND Model and ground truth. Precision for the flooded class reached 100%, indicating that all pixels classified as flooded were indeed flooded, and the absence of false alarms enhances confidence in the model’s flood detection capability. The recall value of 88.3% suggests that while most flooded areas were captured, a small fraction was missed, which is further supported by an F1-score of 93.8%. The specificity for non-flooded regions stood at 100%, confirming that the model perfectly distinguished non-flooded areas. Overall, the Flood map derived from HAND model demonstrates high effectiveness, with robust detection of both flooded and non-flooded regions, though some improvements are needed to minimize the omission of flooded pixels. Based on the analysis, it is identified that the regions around the Thamirabarani river including places such as Eral, Palayakkayal, Athur, Kayalpattinam, Alwarthirunagari, Kurumbur, Sivagalai, Srivaikuntam, Karungukam, Sathankulam, Tiruchendur, Megnanapuram are hight affected by floods.

## Discussions

This study investigated the spatiotemporal characteristics of extreme rainfall events and their impact on flooding in the coastal districts of Kanyakumari, Tenkasi, Tirunelveli, and Thoothukudi, Tamil Nadu, India. While all four coastal districts experience significant variations in rainfall patterns over the past century, the December 2023 event associated with Cyclone Michaung stands out as exceptional. This trend is evident from the data provided by the India Meteorological Department (IMD), highlighting the extreme rainfall on December 17, 2023, which resulted in severe flooding across these districts. The highest rainfall recorded was in Tirunelveli (436.87 mm), followed by Thoothukudi (351.35 mm), Tenkasi (204.81 mm), and Kanyakumari (198.34 mm). The historical rainfall trend analysis (1901–2023) showed a concentration of rainfall in OND (October–December), with increasing frequency of extreme events, particularly in Tirunelveli, which recorded six such events in just a decade. Annual rainfall variability further revealed that while Kanyakumari generally receives higher rainfall, districts like Tirunelveli and Thoothukudi are experiencing greater interannual variability and post-2000 rainfall anomalies, suggesting a heightened dual risk of drought and flood. The OND heatmap and active/break spell analysis confirmed a surge in high-intensity daily rainfall events and a notable absence of prolonged dry spells, highlighting a shift towards concentrated precipitation regimes. This is further corroborated by elevated PCI, PCD, and Seasonality Index (SI) values especially in Tirunelveli and Tuticorin indicating increasingly erratic and seasonally skewed rainfall distributions. The onset and withdrawal analysis of OND rainfall showed delayed and fluctuating seasonal transitions, particularly in Tirunelveli and Kanyakumari, pointing to elongating monsoon durations that complicate agricultural and water management planning. GEV-based frequency analysis across two periods (1901–1960 and 1961–2023) demonstrated rising scale and shape parameters in all districts, reflecting increasing rainfall variability and a growing probability of extreme events. The return period analysis indicates that the rainfall on December 17, 2023, corresponds to a return period of over 100 years for Tirunelveli and Thoothukudi, and 50 years for Kanyakumari and Tenkasi. This indicates that the region experienced an exceptionally high-intensity rainfall event, suggesting a significant shift in the historical rainfall patterns. This trend is concerning as it indicates growing unpredictability in rainfall patterns, which poses challenges for flood management and mitigation efforts. The HAND-based flood inundation mapping effectively identified severely affected areas across the Thamirabarani basin, including Srivaikuntam, Eral, and Kayalpattinam, with an overall accuracy of 92.7% validated against 96 ground truth points. The flooding in urban and peri-urban centers was found to be exacerbated by encroachment of natural floodplains, inadequate drainage infrastructure, and failure of age-old water storage systems like tanks and weirs, many of which breached during the event. These findings collectively indicate that the region’s flood risk is no longer dictated solely by climatological extremes but also by socio-environmental vulnerabilities and outdated infrastructure unable to withstand altered hydrological regimes.

Increasing global warming and climate change across the world are one of the major reasons for the occurrence of such extreme weather events. Global warming is altering atmospheric circulation patterns and increasing sea surface temperatures^30^. This can lead to increased moisture in the atmosphere, making weather systems more conducive to producing extreme rainfall events. The observed rise in rainfall variability across the studied districts aligns with this notion. A warmer atmosphere holds more moisture, and when precipitation occurs, it tends to be more intense and concentrated in specific locations. This can exacerbate flooding risks, especially in low-lying coastal regions.

A study published in Environmental Development in 2016 identified the northern Indian Ocean as a hotspot, experiencing significantly faster warming compared to 90% of the world’s oceans^36^. These hotspots act as early warning signs, allowing scientists to observe the initial impacts of climate change on marine ecosystems. The study highlights the potential of such hotspots by providing early detection of broader trends, assessing the rainfall patterns, and providing early warning for the development of adaptation strategies for a changing climate.

This complex interplay between climatic drivers and land-use change necessitates a rethinking of flood risk governance. Future flood resilience strategies must include district-scale dynamic flood modeling, infrastructure retrofitting based on updated return periods, restoration of traditional water systems, strict zoning regulations, and community-driven early warning systems. The study not only documents the emerging pattern of compound climate risks in semi-arid monsoon regions but also emphasizes the urgent need for integrated, anticipatory, and adaptive flood management strategies to safeguard vulnerable landscapes and communities across southern Tamil Nadu.

## Conclusions

December 2023 floods in the southern Tamil Nadu districts of Kanyakumari, Tenkasi, Tirunelveli, and Thoothukudi serve as a stark reminder of the growing threat posed by extreme weather events. This event, triggered by Cyclone Michaung, brought exceptionally high rainfall to the region, exceeding a 100-year return period in Tirunelveli and Thoothukudi. The analysis of historical rainfall data reveals a concerning trend of increasing variability and extremity in rainfall patterns across these districts. While Kanyakumari exhibits slight fluctuations, Tenkasi, Tirunelveli, and Thoothukudi show a consistent rise in the scale factor, indicating a growing potential for extreme rainfall events. This trend aligns with the well-established effects of climate change, where a warmer atmosphere holds more moisture, leading to more intense and concentrated precipitation events.

This study has demonstrated the increasing frequency and intensity of extreme rainfall events and their direct implications for flood risk in the coastal districts of southern Tamil Nadu namely, Kanyakumari, Tenkasi, Tirunelveli, and Thoothukudi. Through detailed historical analysis using IMD rainfall data (1901–2023), seasonality metrics (PCI, PCD, SI), GEV-based frequency modeling, and HAND-based flood mapping, it is evident that the region is undergoing a hydrometeorological regime shift. The December 2023 event, linked to Cyclone Michaung, resulted in record-breaking rainfall intensities and widespread flooding, with Tirunelveli and Thoothukudi surpassing 100-year return periods. Rainfall clustering during OND months, a rise in scale and shape parameters, and the absence of break spells indicate a transition toward shorter, more intense rain episodes. The HAND model provided a robust estimation of inundation extent, but also underscored the vulnerability of existing infrastructure and urban systems. These findings confirm that both climatic and anthropogenic stressors ranging from oceanic warming to land-use mismanagement are intensifying flood hazards in the region. As such, flood events can no longer be considered isolated or random; they are symptomatic of evolving climate dynamics and infrastructural inadequacies.

To build predictive capacity and future resilience, it is imperative to transition from static flood risk assessments to scenario-based dynamic flood modeling frameworks. We recommend the integration of the Variable Infiltration Capacity (VIC) model for large-scale, grid-based hydrological simulation to estimate runoff and soil moisture responses to extreme precipitation. This hydrological output can then be coupled with HEC-RAS for 2D floodplain hydraulic modeling and SWMM (Storm Water Management Model) for detailed urban drainage and stormwater simulation. Together, these models can generate high-resolution flood inundation scenarios under both current and future climatic conditions. Additionally, to project flood risks in the context of climate change, we propose the use of downscaled CMIP6 climate datasets, particularly under shared socioeconomic pathways (SSPs), to simulate rainfall extremes and assess future return periods. Coupling CMIP-driven rainfall projections with the VIC–HEC-RAS–SWMM modeling chain will enable researchers and policymakers to delineate flood-prone zones under a range of scenarios and guide infrastructure adaptation planning. Such a multi-model, climate-informed approach is essential for developing robust early warning systems, resilient infrastructure design standards, and district-level flood mitigation strategies tailored to the complex hydroclimatic realities of southern Tamil Nadu.

## Data Availability

The dataset utilized/analyzed during the current study will be available from the corresponding author upon request.
